# Biochemical and Proteomic Characterization, and Pharmacological Insights of Indian Red Scorpion Venom Toxins

**DOI:** 10.3389/fphar.2021.710680

**Published:** 2021-09-28

**Authors:** Bhabana Das, Anthony J. Saviola, Ashis K. Mukherjee

**Affiliations:** ^1^ Department of Molecular Biology and Biotechnology, School of Sciences, Tezpur University, Tezpur, India; ^2^ Department of Biochemistry and Molecular Genetics, University of Colorado Anschutz Medical Campus, Aurora, CO, United States; ^3^ Institute of Advanced Study in Science and Technology, Guwahati, India

**Keywords:** Indian red scorpion, venom composition, pathophysiology of scorpion sting, catecholamines, therapy against Mesobuthus tamulus scorpion sting

## Abstract

The Indian red scorpion (Mesobuthus tamulus) is one of the world’s deadliest scorpions, with stings representing a life-threatening medical emergency. This species is distributed throughout the Indian sub-continent, including eastern Pakistan, eastern Nepal, and Sri Lanka. In India, Indian red scorpions are broadly distributed in western Maharashtra, Saurashtra, Kerala, Andhra Pradesh, Tamil Nadu, and Karnataka; however, fatal envenomations have been recorded primarily in the Konkan region of Maharashtra. The Indian red scorpion venom proteome comprises 110 proteins belonging to 13 venom protein families. The significant pharmacological activity is predominantly caused by the low molecular mass non-enzymatic Na^+^ and K^+^ ion channel toxins. Other minor toxins comprise 15.6% of the total venom proteome. Indian red scorpion stings induce the release of catecholamine, which leads to pathophysiological abnormalities in the victim. A strong correlation has been observed between venom proteome composition and local (swelling, redness, heat, and regional lymph node involvement) and systemic (tachycardia, mydriasis, hyperglycemia, hypertension, toxic myocarditis, cardiac failure, and pulmonary edema) manifestations. Immediate administration of antivenom is the preferred treatment for Indian red scorpion stings. However, scorpion-specific antivenoms have exhibited poor immunorecognition and neutralization of the low molecular mass toxins. The proteomic analysis also suggests that Indian red scorpion venom is a rich source of pharmacologically active molecules that may be envisaged as drug prototypes. The following review summarizes the progress made towards understanding the venom proteome of the Indian red scorpion and addresses the current understanding of the pathophysiology associated with its sting.

## A Brief Overview of Venomous Scorpions With Particular Reference to Indian Red Scorpion

Animal venoms are complex secretions consisting mainly of bioactive proteins and peptides (Fry et al., 2009; Casewell et al., 2013) that primarily serve as a chemical means of protection and subduing prey. Venom has evolved independently throughout the animal kingdom (Casewell et al., 2013; Suranse et al., 2018); consequently, it is present in all major animal lineages (Holford et al., 2018). All of the 1,500 extant scorpion species are venomous. While human envenomation by most results in only minor reactions, approximately 30 scorpions are considered medically significant (Table 1) (Bawaskar and Bawaskar, 2012). Severe and sometimes fatal envenomations have been documented from stings by Buthidae, Hemiscorpiidae, and Scorpionidae families (White, 2016) in Latin America, North Africa, the Middle East, and India (Reddy, 2013). Currently, 86 scorpion species have been described throughout India; however, only the Indian red scorpion (*Mesobuthus tamulus*), which belongs to the family Buthidae, and the Indian black scorpion (*Heterometrus swammerdami*, formerly *Palamneus gravimanus*) of the Scorpionidae family, pose a significant threat to humans– primarily young children, elderly, and immuno-compromised individuals (Tiwari and Deshpande, 1993; Badhe et al., 2007; Quintero-Hernandez et al., 2013; Reddy, 2013; Ortiz et al., 2015; Santos et al., 2016; Das et al., 2020). Limited clinical reports suggest that the venom of the Indian red scorpion exhibits higher toxicity compared to Indian black scorpion venom (Erfati, 1978; Bawaskar and Bawaskar, 1998; Madhavan, 2015; Senthilvelan et al., 2015), and as a consequence, urgent medical attention may be required following a sting. The potent toxicity of Indian red scorpion venom is attributed to the abundance of potassium channel toxins targeting the central nervous and cardiovascular systems (discussed below). This venom phenotype is also seen in other medically important scorpion species, such as the Iranian scorpion (*Hemiscorpius lepturus*) of the Hemiscorpiidae family (Prendini, 2000), and *Pandinus imperator* (Scorpionidae family), which is endemic to West Africa.

**TABLE 1:** List of some of the most dangerous and deadly scorpion species distributed across the world.

Common name	Scientific name	Geographical distribution	References
Sahara scorpion	*Androctonus australis* (Ewing, 1928)	North Africa and Middle East	Touloun et al. (2001)
Arabian fat-tailed scorpion	*Androctonus crassicauda* (Olivier, 1807)	Turkey, Middle East and North Africa	Radmanesh, (1990); Dittrich et al. (1995)
Yellow scorpion	*Buthus occitanus* (Amoreux, 1789)	countries bordering Mediterranean and Middle East	Ghalim et al. (2000)
Death stalker	*Leiurus quinquestriatus* (Ehrenberg, 1829)	North Africa and Middle East	Dittrich et al. (1995)
South African fattail scorpion	*Parabuthus transvaalicus* (Purcell, 1899)	South Africa	Bergman (1997)
Trinidad thick- tailed scorpion	*Tityus trinitatis* (Pocock, 1897)	Trinidad and Venezuela	Daisley et al., 1999
Brazilian scorpion	*Tityus bahiensis* (Perty, 1833)	Brazil, Argentina	Bucaretchi et al. (1995)
Arizona bark scorpion	*Centruroides sculpturatus* (Ewing, 1928)	California, New Mexico, Arizona and Baja California	Chowell et al. (2006); Dehesa-Dávila and Possani, 1994
Indian red scorpion	*Mesobuthus tamulus* (Fabricus, 1798)	India	Kovarik, (2007)

Given the medical threat of the Indian red scorpionand the recent characterization of its venom proteome (Das et al., 2020), the following review aims to highlight the current understanding of the venom proteome composition, the epidemiology of Indian red scorpion sting, and correlate venom phenotype to the pathophysiological symptoms observed following envenomation. Lastly, we address some of the different treatment regimens utilized by clinicians to treat scorpion stings. Searches of published reports were conducted with public databases (MEDLINE, Scopus) using the search engines-Science Direct (https://www.sciencedirect.com/), Google Scholar (https://scholar.google.com/), and PubMed (pubmed.ncbi.nlm.nih.gov). The different search words were “Indian red scorpion venom,” “Toxins and Indian red scorpion venom,” “epidemiology of Indian red scorpion sting,” “Proteomic analysis and Indian red scorpion venom,” “Pathophysiology of Indian red scorpion sting,” “*Mesobuthus tamulus*,” and “Treatment of scorpion sting.”

## Geographical Distribution, Epidemiology, and Clinical Symptoms of Sting

**FIGURE 1:**
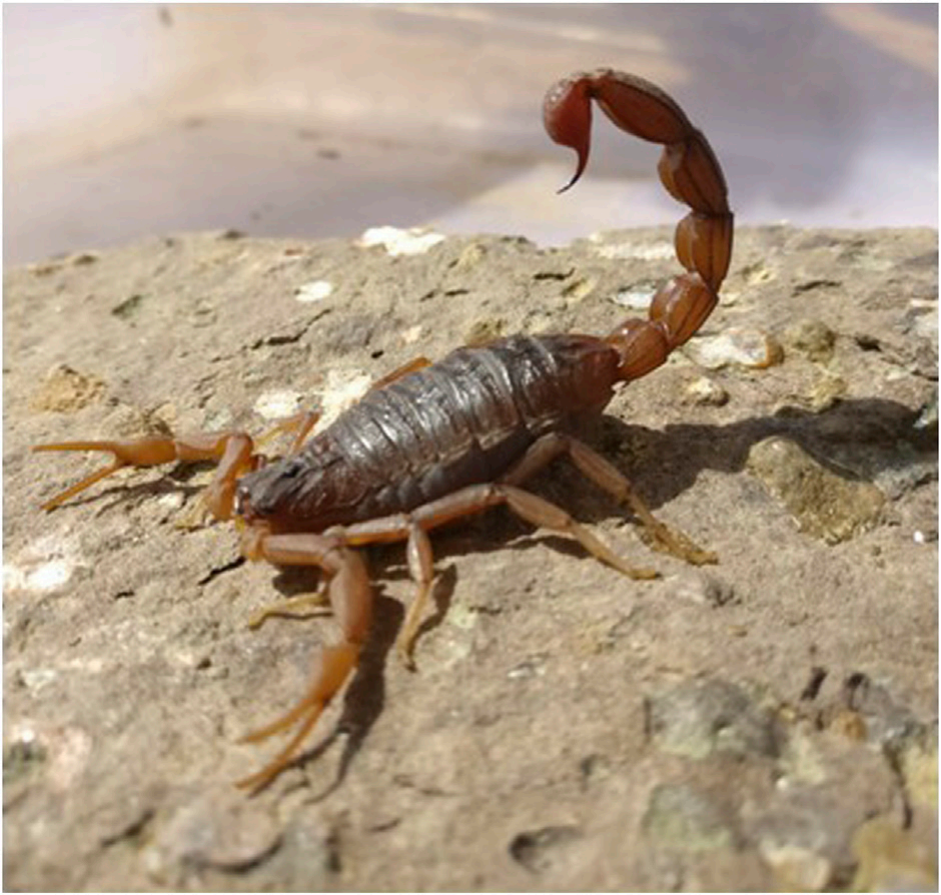
Indian red scorpion *(Mesobuthus tamulus)* (Photo courtesy, Dr. M. V. Khadilkar).

The evolutionary history of scorpions is represented by a continuous fossil record that dates back to the Telychian Stage (Silurian, Llandovery) (Dunlop, 2010; Dunlop and Selden, 2013). Evidence suggests that scorpions have existed on earth for over 430 million years and first appeared as an aquatic organism during the Silurian period (Dunlop and Selden, 2013). The present-day Indian red scorpion ranges in size from 2 to 3.5 inches (5–9 cm) in length (Kovařík, 2007), has red pedipalps (claws), a tail, legs, and body covered in khaki-colored cuticles (Figure 1). These nocturnal predators are endemic to the Indian sub-continent. They are rarely found outside Eastern Nepal (Bhadani et al., 2006), Eastern Pakistan (Kovařík, 2007), or Sri Lanka (Kularatne et al., 2015).

In India, morbidity and mortality due to scorpion stings frequently occur in western Maharashtra, Saurashtra, Kerala, Andhra Pradesh, Tamil Nadu, and Karnataka. A case study involving 141 children admitted to Government Raja Mirasdhar Hospital (Thanjavur, southern India) with a real Indian red scorpion sting, demonstrated that childrenbetween 1–3 and 7–12 years of age exhibited the following: most adverse effects to envenomation. Eight patients displayed priapism and five patients up to 6 years. One patient older than 6 years exhibited pulmonary edema, a fatal and life-threatening sting effect (Yuvaraja et al., 2019). Records from a tertiary care and teaching hospital in southern India showed that 50 patients experiencing Indian red scorpion sting demonstrated dyspnoea (*n* = 13, 26%), chest pain (*n* = 9, 18%), vomiting (*n* = 6, 12%), sweating (*n* = 5, 10%), nausea (*n* = 3, 6%), priapism (*n* = 7, 14%) and piloerection (*n* = 6, 12%) (Madhavan, 2015).

An epidemiological study conducted in Mahad (200 km south of Mumbai, Western India) from 1984 to 1995 also showed that children <16 years tend to respond more poorly to Indian red scorpion sting (Bawaskar and Bawaskar, 1998); out of the 293 patients, six deaths were reported before hospital arrival. Patients were further divided into three broad groups based on the clinical symptoms- i) 111 (38%) patients exhibited hypertension within 1–10 h (mean 3.5 h), ii) 87 (30%) patients with tachycardia reported within 1–24 h (mean 6.7 h), and iii) 72 (24.5%) patients with pulmonary oedema reported within 6–24 h (mean 8 h) post scorpion sting (Bawaskar and Bawaskar, 1996; Bawaskar and Bawaskar, 1998).

A 14 year old healthy male from the Babaganj region of Northern India developed cardiac and gastrointestinal complications following an Indian red scorpion sting on the right big toe (Agrawal et al., 2015). Twenty-three Indian red scorpion stings have been documented in three localities of Jaffna, Sri Lanka, consisting of 13 (57%) males and 10 (43%) females. While the mean age was 30 years (Kularatne et al., 2015), 5 (22%) cases were children below 12. Upon admission to the hospital, all patients had evidence of either local or systemic manifestations envenoming (Kularatne et al., 2015; Ratnayake et al., 2016). Thirty-three scorpion stings were reported at Rims Teaching Hospital, Raichur, Karnataka, India, from 2009 to 2014, of which 22 were from the Indian black scorpion and 11 from the Indian red scorpion. The patients exhibited bradycardia, drowsiness, cutaneous manifestations, hypotension, and hypertension (Rajashekhar and Mudgal, 2017).

## Diversity of Indian Red Scorpion Venom

**FIGURE 2:**
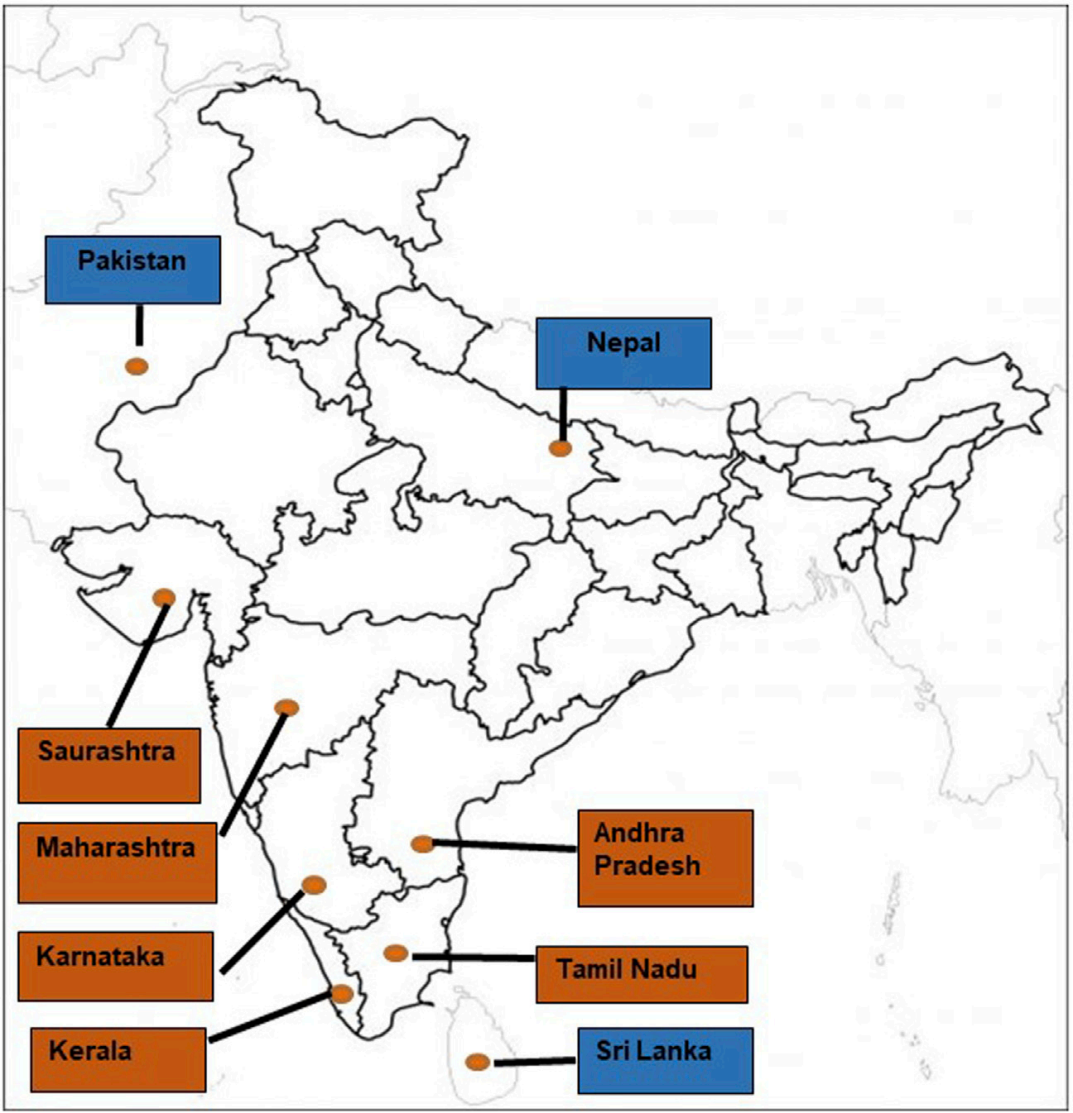
The geographical distribution of the Indian red scorpion throughout the Indian sub-continent [Brown fill: Indian states; Blue fill: neighbouring countries of India].

Geographical variation in sting severity has been reported in India (Reddy, 2013; Suranse et al., 2019) (Figure 2) and is a likely consequence of variation in population genetic structure, which drives phenotypic differences in venom composition (Newton et al., 2007). Several populations of Indian red scorpions collected from eight locations in Maharashtra (Bhate plateau, Sangameshwar, Jejuri, Shindavane, Pashan, Alandi, Kalyan, and Jalna) exhibited moderate genetic variation, with regression analysis suggesting that the genetic distance of subspecies increases by 0.006% (95%CI: 0.003–0.010%) per Kilometre of geographical separation (Suranse et al., 2017). It has also been suggested that genetic structure correlates to climatic differences in precipitation, specifically high, moderate, and low rainfall areas (Suranse et al., 2017), associated with differences in venom phenotype. For example, significant variation in the expression of venom peptides was observed between Indian red scorpions collected from the Konkan region of Maharashtra and the semi arid Deccan plateau (Newton et al., 2007). In addition, anecdotal reports suggest that stings from Indian red scorpions of the Konkan region on the western side of the Western Ghats are more severe than stings from populations on the eastern side of the Western Ghats. These differences are likely due to variations in venom peptide composition between the two populations (Newton et al., 2007). While other factors may contribute to the pathophysiology of the sting, these have not been addressed in the literature (Bawaskar and Bawaskar, 1992; Kankonkar et al., 1998; Murthy and Zare, 1998; Newton et al., 2007). Intra-specific venom variation has also been demonstrated between Indian red scorpions from Western India (Ratnagiri, Chiplun, and Ahmednagar) and Southern India Chennai by sodium dodecyl polyacrylamide gel electrophoresis (SDS-PAGE) (Badhe et al., 2006). Mice injected with equal concentrations of Indian red scorpion venom obtained from the above geographical regions showed significant variation in their blood sodium levels (Badhe et al., 2007). While in-depth analyses of Indian red scorpion venoms from different areas of the Indian sub-continent are currently lacking, this data would help uncover the geographical impact onvenom composition. Although venom variation has been shown to result in differences in sting severity and symptoms for scorpions from different regions of the world (Abroug et al., 2020), a detailed description of this topicis beyond the scope of the current review.

## Characterization of Venom and Analyses of Sequence-Structure-Functional Impacts

### Biochemical and Proteomic Characterization

Scorpion venomis a cocktail of enzymatic and non-enzymatic proteins with the latter classified into two additional categories based on their number of amino acids; i) short toxins are comprised of 30–40 amino acids; and ii) long toxins have 60–70 amino acids (Srairi-Abid et al., 2019). Non-enzymatic toxins can be divided into four groups based on their biological functions and pharmacological activity, namely Na^+^, K^+^, Ca^2+^, and Cl^−^ channel toxins (Possani et al., 1999; Tytgat et al., 1999; de la Vega and Possani, 2004). The last decade has seen an expansion of research techniques utilized to identify, characterize, and quantify the venom composition of venomous animals. Traditional approaches have relied on biochemical analyses of venom enzymes and venom profiling by SDS-PAGE and gel filtration chromatography. However, more recently, these techniques have been coupled with high-throughput genomic, transcriptomic, and proteomics approaches to provide a more profound and more comprehensive analyses of a species venom (Gutiérrez et al., 1995; Calvete et al., 2009; de la Vega et al., 2010; Abdel-Rahman et al., 2013; Mukherjee et al., 2016; Santibáñez-López et al., 2016; Calvete, 2017; Kalita et al., 2018; Saviola et al., 2020). Several studies have also drawn a good correlation between venom composition with toxicity and pathophysiology of sting (Zelanis and Tashima, 2014; Chanda and Mukherjee, 2020; Manuwar et al., 2020).

Recently, our laboratory utilized liquid chromatography-mass spectrometry (LC-MS/MS)-based proteomics combined with biochemical and *in vitro* pharmacological activity assays to characterize the venom composition of the Indian red scorpion (Das et al., 2020). Proteomic analysis identified 110 proteins and polypeptides belonging to 13 protein families. The venom had a preponderance of ion channel toxins (Na^+^ and K^+^ channels affecting toxins). Other minor venom components are serine protease-like protein, serine protease inhibitor, antimicrobial peptide, hyaluronidase, makatoxin, lypolysis potentiating peptides, neurotoxin affecting Cl^−^ channels, parabutoporin, Ca^2+^ channel toxins, bradykinin potentiating peptides, HMG CoA reductase inhibitor, and several other toxins with unknown pharmacological activity (Das et al., 2020, Supplementary Table S1). Further, the low molecular weight insect-selective toxins BtTx3 (3,796 Da) and ButaIT (3,856.7 Da) were identified. These toxins can be developed as insecticidal agents against lepidopteran insect species (Wudayagiri et al., 2001; Dhawan et al., 2002).

Indian red scorpion venom did not show activity for any of the tested enzymes (phospholipase A_2_, L-amino acid oxidase, adenosine tri-, di-, and monophosphatase, hyaluronidase, metalloproteinase, and fibrinogenolytic), was devoid of *in vitro* hemolytic activity, and also failed to interfere with blood coagulation and platelet modulation (activation or deaggregation) under *in vitro* conditions.The 3D structure of some of the toxins deposited in UniProt is shown in Supplementary Table S2. The occurrence of several other venom toxins from different *Mesobuthus* and *Heterometrus* species also found throughout the Indian subcontinent are shown in Supplementary Table S3.

### Sturture-Function Analysis of Scorpion Toxins

Scorpion venoms contain numerous polypeptides cross-linked via three to four disulfide bridges that exert various physiological and pharmacological activities by targeting ion channel (s) function (Rochat et al., 1979; Zlotkin et al., 1991; Gordon et al., 1992). Regardless of the diverse primary structures, the majority of scorpion toxins have an identical Csαβ (cysteine-stabilized α/β motif) fold (Fontecilla-Camps et al., 1988). Further, Na^+^ channel toxins have been divided into mammalian and insect toxins, with the former sub-divided into α- and β- toxins (Jover et al., 1980; Couraud et al., 1982) and the latter sub-classified into depressant, excitatory, and α- insect toxins (Zlotkin et al., 1995). Several scorpion toxins that exclusively target Na^+^ and K^+^ channels have been studied extensively concerning their structure, mode of action, and pharmacological properties (Miller et al., 1985; Zlotkin et al., 1991; Delepierre et al., 1997). Little attention has been paid to identifying and purifying toxins from Indian red scorpion venom to develop different drug prototypes. However, the sequences of a few purified toxins have been determined. For example, ButaIT, is a novel short lepidopteran-selective toxin with 37 amino acids cross-linked by disulfide bridges and eight cysteine residues; it shares sequence homology with other fast toxins Peptide I, neurotoxin P2, Lqh-8/6, chlorotoxin, insectotoxin I5A, insect toxin 15, and insectotoxin I1. Three-dimensional structural modelling of this toxin revealed that similar to other scorpion toxins, ButaIT contains an α-helix and a β-sheet. Moreover, this toxin showed high target specificity towards Heliothis virescens, *a notorious budworm* on the cotton crop (Wudayagiri et al., 2001). Notably, the proteomic analysis demonstrated that Bukatoxin and Makatoxin represent approximately 2.3% of *M. tamulus* venom proteome (Das et al., 2020).

Although the structure-function relationship of these toxins from Indian red scorpion venom has not been elucidated; the functional site of Bukatoxin from *Buthus martensii karch* venom activates the Na^+^ channel in nitrergic inhibitory fibers, resulting in the neuronal release of nitric oxide (NO) (Gibson and McFadzean, 2001; Srinivasan et al., 2001; Reis et al., 2019). Bukatoxin shares 78 and 72% structural similarity with neurotoxin X from *Mesobuthus eupeus* venom (Grishin et al., 1979) and neurotoxin IV from *Leiurus quinquestriatus quinquestriatus* venom (Kopeyan et al., 1985), respectively. Similarly, Makatoxin I from venom of *Buthus martensii karch* contains 64 amino acids with eight half cystein residues, has a short J loop (cys- 16 to cys-22) and a long B loop (cys-36 to cys-46) (Gong et al., 1997) and exhibits 78 and 81% structural similarity with Bot I (Vargas et al., 1982) and Bot II (Gregoire and Rochat, 1983) toxins (both from *Buthus occictanus tunetanus* venom), respectively. Further, Makatoxin I shows 55–77% similarity with Lqq IV and Lqq III from *Leiurus quinquestriatus quinquestriatus* venom (Kopeyan et al., 1985; Kopeyan et al., 1993)*.* The nitrergic action of Makatoxin I causes a release of NO that mediates a relaxant response in rat precontracted anococcygeus muscle (ACM) (Gong et al., 1997).

## Pharmacological Targets of Indian Red Scorpion Toxins

Indian red scorpion venom is quite toxic towards mammals. The median lethal dose (LD50, s.c injection) against the juvenile and adult rats has been estimated at 1.3 ± 0.14 and 2.2 ± 0.21 mg/kg, respectively (Tiwari and Deshpande, 1993). Consequently, the Indian red scorpion sting represents a significant medical threat throughout its geographical range, including in India. The potent toxicity towards mammals is due to a predominance of neurotoxins (Das et al., 2020) that target the functionality of voltage-gated Na+ and K+ channels, including calcium-activated K^+^ media, which ultimately leads to the various clinical symptoms in different regions of India and Sri Lanka (Table 2).

**TABLE 2:** Comparative list of the pharmacological effects induced by toxins from Indian red scorpion venoms of different geographical regions.

Sl. No	Pharmacological effects	Responsible toxins	Geographical region				References
			Southern India	Western India	Northern India	Sri Lanka	
1	Vomiting, nausea	Na^+^ channel toxin (α neurotoxin)	YES (Yuvaraja et al., 2019)	YES (Bawaskar and Bawaskar, 1998)	YES (Agrawal et al., 2015)	Not known	Jimenez et al. (2008)
2	Sweating	Na^+^ channel toxin (α neurotoxin)	YES (Yuvaraja et al., 2019)	YES (Bawaskar and Bawaskar, 1998)	YES (Agrawal et al., 2015)	YES (Kularatne et al., 2015)	Jimenez et al. (2008)
3	Salivation	Na^+^ channel toxin (α neurotoxin)	Not known	YES (Bawaskar and Bawaskar, 1998)	Not known	YES (Kularatne et al., 2015)	Jimenez et al. (2008)
4	Bradycardia, hyperkalamia, vasoconstriction	Na^+^ channel toxin (α neurotoxin)	Not known	Not known	Not known	Not known	Jimenez et al. (2008); Tiwari and Deshpande, (1996)
5	Tachycardia	Na^+^ channel toxin (α neurotoxin)	YES (Bawaskar and Bawaskar, 1998; Madhavan, 2015; Yuvaraja et al., 2019)	YES (Bawaskar and Bawaskar, 1996)	Not known	YES (Kularatne et al., 2015)	Jimenez et al. (2008)
6	Pulmonary oedema	Na^+^ channel toxin (α neurotoxin)	YES (Bawaskar and Bawaskar, 1998; Madhavan, 2015; Yuvaraja et al., 2019)	YES (Bawaskar and Bawaskar, 1996)	Not known	Not known	Jimenez et al. (2008)
7	Chest pain	Na^+^ channel toxin (β neurotoxin)	YES (Yuvaraja et al., 2019)	Not known	YES (Agrawal et al., 2015)	Not known	Stevens et al. (2011)
8	Breathlessness, cough	Na^+^ channel toxin (β neurotoxin)	Not known	Not known	YES (Agrawal et al., 2015)	Not known	Stevens et al. (2011)
9	Cardiac arrythmias	K^+^ channel toxin	Not known	YES (Bawaskar and Bawaskar, 1998)	Not known	Not known	Ravens and Cerbai, (2008)
10	Hypotension	Bradykinin potentiating peptide	YES	Not known	Not known	YES (Kularatne et al., 2015)	Ianzer et al. (2004)
11	Hypertension	Ca^2+^ channel toxin	YES (Yuvaraja et al., 2019)	YES (Bawaskar and Bawaskar, 1996)	Not known	YES (Kularatne et al., 2015)	Fan et al. (2015); Touyz et al. (2018)
12	Priapism	Bukatoxin and Makatoxin	YES (Madhavan, 2015; Yuvaraja et al., 2019)	YES (Bawaskar and Bawaskar, 1998)	Not known	Not known	Gibson and McFadzean (2001)
13	Piloerection	Not characterized	YES (Yuvaraja et al., 2019)	Not known	Not known	YES (Kularatne et al., 2015)	—
14	Myocarditis	Na^+^ channel toxin (α neurotoxin)	YES (Bawaskar and Bawaskar, 1998; Madhavan, 2015; Yuvaraja et al., 2019)	Not known	Not known	Not known	Jimenez et al. (2008)

Proteomic analysis revealed that Na^+^ and K^+^ channel toxins are prominent in Indian red scorpion venom. These toxins are likely responsible for enhancing the release of neurotransmitters, either by slowing the inactivation of Na^+^ channels or blocking the K^+^ channels (Narahashi et al., 1972; Rowan et al., 1992; Vatanpour et al., 1993). Na^+^ channel toxins are further classified as α- and β-neurotoxins and can interfere with the function of the nervous system by modulating Na^+^ channel activity in nerve cells (Stevens et al., 2011). The α-toxin binds to site 3 receptors of Na^+^ channel, where it blocks the inactivation and prolongs the action potential (Catterall, 1976; Catterall, 1986; Couraud et al., 1982). On the other hand, the β-toxin binds to site 4 of Na+ channel and shifts the voltage activation towards more negative potentials, leading to spontaneous and repetitive firing (Couraud et al., 1982; Pinter et al., 1999).

Tamapin, a 3,459.1 Da toxin purified from Indian red scorpion venom, selectively blocks small conductance Ca^2+^-activated K^+^ (SK) channels expressed in the central nervous system. Tamapin has 31 amino acids and exhibits moderate sequence similarity to scyllatoxin (77%) and PO5 (74%), two SK channel blockers from *Leiurus quinquestriatus,* and *Androctonus mauretanicus* venoms, respectively. Further, the C-terminal tyrosine residue of tamapin is amidated, which likely impacts the pharmacological properties and potency of the toxin (Pedarzani et al., 2002). Tamulustoxin, another peptide toxin from Indian red scorpion venom, exhibits a slow time-dependent inactivation of the K^+^ channel that produces a functional effect on the prolonged depolarization or repetitive firing of action potentials (Strong et al., 2001). Notably, scorpion venom toxins that block Na^+^ and K^+^ channels mediate the synergistic effects responsible for the intense and persistent depolarization of the autonomic nerves, causing a massive release of autonomic neurotransmitters that evokes the “autonomic storm” response (Gwee et al., 2002). Scorpion venom toxins that target Ca^2+^ channels inhibit the contraction of pulmonary artery smooth muscle cells by decreasing intracellular calcium and causing pulmonary hypertension (Fan et al., 2015; Touyz et al., 2018).

Interestingly, Indian red scorpion venom lacked enzymatic activity, even though mass spectrometry identified several enzymes (Das et al., 2020). Proteomic analysis identified a serine protease-like protein and a hyaluronidase enzyme comprising 2.9 and 2.2% of venom, respectively. However, crude venom did not show serine protease activity or influence clotting time of platelet poor plasma; hyaluronidase activity was also absent (Das et al., 2020). Interestingly, hyaluronidase activity was demonstrated by some other scorpion species such (*Heterometrus swammerdami*/*Palamneus gravimanus* (Morey et al., 2006), *Hemiscorpius lepturus* (Seyedian et al., 2010), and *Heterometrus fuvipes* (Ramanaiah et al., 1990), and Strong et al. (2001) also reported enzymatic activities from scorpion venoms. It is also possible that the functionality of these enzymes differs from the conventional snake venom serine proteases and hyaluronidases, resulting in differences in substrate specificity between some snake and scorpion venom enzymes (Thakur and Mukherjee, 2015; Cid-Uribe et al., 2020; Das et al., 2020). Another possibility is that the serine protease-like protein and the hyaluronidase enzyme do not show *in vitro* enzyme activity due to their relatively low abundance in Indian red scorpion venom (Das et al., 2020). It is noteworthy that while hyaluronidase is a non-toxic enzyme, it enhances the diffusion rate of venom into the victim’s tissue and thus enhances the local systemic envenomation (Morey et al., 2006). Moreover, the presence of phospholipase A_2_ (MtPLA_2,_19 kDa) in Indian red scorpion venom was reported by Hariprasad et al. (2009). However, the activity of this enzyme was not detected, possibly due to its low abundance in the venom proteome (Das et al., 2020). PLA_2_ has been reported in the venom of other scorpions such as *Anuroctonus phaiodactylus* (Phaiodactylipin, 19.1 kDa) (Valdez-Cruz et al., 2004), *Hemiscorpius lepturus* (Hemipilin 1 and 2, 15 kDa) (Jridi et al., 2015, 2017), *Heterometrus fulvipes* (HfPLA_2_, 16 kDa) (Ramanaiah et al., 1990), *Heterometrus laoticus* (HmTx, 14 kDa) (Incamnoi et al., 2013), *Pandinus imperator* (IpTxi, 15 kDa) (Zamudio et al., 1997), *Scorpio maurus* (phospholipin and Sm-PLGV, 14.8 and 15.15 kDa, respectively) (Conde et al., 1999; Louati et al., 2013; Krayem et al., 2018). PLA_2_s exhibit a broad spectrum of pharmacological activities, ranging from myotoxicity, neurotoxicity, inflammatory, hemolytic, anticoagulant, anti-microbial to anti-tumoral activities (Krayem and Gargouri, 2020).

## Elucidation of Mechanism of Toxicity

The mechanism of scorpion venom-mediated toxicity in an envenomed human is briefly discussed below.

### Myocarditis, Bradycardia, and Hypotension

Indian red scorpion sting is often characterized by myocarditis, bradycardia, and hypotension (Bawaskar, 1982). The binding of scorpion venom neurotoxins to Na+ ion channels promotes membrane depolarization which triggers the release of catecholamines (Rowan et al., 1992; Jimenez et al., 2008; Stevens et al., 2011). Continuous elevation of these adrenalin hormones can downregulate β-adrenergic receptors, which are critical for the overall regulation of cardiac function (Wachter and Gilbert, 2012). This effect can diminish myofibrils’ role and cause myocarditis and bradycardia by lowering the number of contracting units, reducing the heart’s pumping ability (Singh and Deshpande, 2005; Kassim et al., 2008).

### Tachycardia and Hypertension

Tachycardia is correlated with hypertension and may induce cardiovascular risk. As mentioned above, catecholamines released following scorpion envenomation can play a crucial role in initiating cardiac disorder by activating the β-receptors in the heart. The continuous release of endogenous and exogenous catecholamines post Indian scorpion sting causes an increase in spontaneous diastolic depolarization on cardiac fibers, which elevates the heart rate that leads to tachycardia or tachyarrhythmia (Reddy et al., 2017).

### Pulmonary Edema

The fluid movement across the pulmonary capillary membrane can be described through a general transport equation named Starling’s equation (Starling, 1896). The elevated release of catecholamines as a consequence of scorpion sting can produce a profound shift influid movement, resulting in a collection of fluid in the extravascular tissue of the lung (pulmonary oedema) (Erdmann et al., 1975). Pulmonary oedema is a common clinical symptom of Indian red scorpion sting (Bagchi, and Deshpande, 1998).

### Priapism

Bukatoxin and makatoxin are two α-neurotoxins from Indian red scorpion venom (Das et al., 2020) that cause a persistent activation of Na^+^ channel in nitrergic inhibitory fibers resulting in the release of NO (Gibson and McFadzean, 2001; Gwee et al., 2002; Reis et al., 2019). Upon entering the sting victim’s body, these neurotoxins stimulate the production of acetylcholine, which binds to endothelial cell receptors following activation of NO synthase to produce NO, which then passes through the smooth muscle cell to activate guanylate cyclase leading to the synthesis of cGMP. After that, cGMP activates protein kinase G, which acts as a vasodilator of smooth muscle causing priapism (unwanted, prolonged, painful penile erection), a common clinical symptom primarily observed in children post scorpion sting (Bawaskar, 1982; Hofmann et al., 1992; Lincoln and Cornwell, 1993; Lohmann et al., 1997). According to Bawaskar (1982), the chances of developing cardiac manifestations at a later stage of priapism are very high.

## A Brief Account on Treatment of Scorpion Sting in India

As Indian red scorpion sting can be lethal, significant effort must be directed towards understanding the associated pathophysiological symptoms and adequately treating the envenomated victim. Despite advances in understanding pathophysiology and therapy, mortality remains high in many rural areas, mainly due to inefficient access to medical facilities (Reddy, 2013). Following a scorpion sting, patients should be observed for 24 h irrespective of the species involved. For the cases of severe envenomation, therapeutic efforts should be directed towards treating the over stimulated autonomic nervous system and correcting hypovolemia. Different treatment strategies utilized for Indian red scorpion sting are summarized in Table 3. Depending upon the severity of the sting, antivenom availability, and access to proper medical facilities, a single or a combination of treatment methods may be administered (Bawaskar and Bawaskar, 2011).

**TABLE 3:** Different treatment regimes utilized for scorpion sting.

Types of treatment	Treatment methods	Mode of action of drug/auxiliary treatment	References
Treatment of local symptoms	•Ice packs	•Ice pack can reduce blood flow and nerve activity which reduces pain	Freire-Maia, et al. (1994); Kokki, (2003); Bahloul et al. (2013)
	•lignocaine (without adrenaline) using ring block	—	
	•Dipyrone, a pyrazolone derivative	—	
	•local anaesthesia is applied with lidocaine	—	
	•oral diazepam and non-steroidal anti-inflammatory drugs (NSAIDs)	•The main mechanism of action of NSAID is the inhibition of cyclooxygenase (COX)	
Treatment of shock	•Elevation of the foot end of bed to maintain cerebral circulation in cases of peripheral circulatory failure	Metaclopramide can prevent nausea and vomiting due to its action at D_2_ receptor in central nervous system	Freire-Maia, et al. (1994); Abroug et al. (1997); Rang, (2003)
	•Metoclopramide, oral and parenteral fluids		
	•Intravenous glucose, normal saline		
	•100 mg of hydrocortisone after every 4 h		
Treatment by prazosin	Prazosin is administered either orally or sometimes given through a nasogastric tube if the patient is vomiting	Prazosin can control the arterial blood pressure and other pharmacological effects by blocking the alpha1 receptors in muscle cell and cause vasodilatation of the blood vessel	Reader et al. (1987); Reddy, (2013)
Treatment by insulin	Administration of insulin with or without alpha blocker and sodium bicarbonate	Insulin can neutralize the effect of catecholamines favouring glucose uptake, enormous boost in glycogen content in the liver, skeletal and cardiac muscles, and promote lipogenesis in animals with scorpion sting (Yugandhar et al., 1999). Moreover, administration of insulin along with an alpha blocker and sodium bicarbonate can diminish the rate of arrhythmias and also reverse the metabolic and electrocardiographic changes after scorpion envenomation (Radha et al., 1988)	Yugandhar et al. (1999)
Specific treatment by antivenom	Administration of PSVPL, Haffkine scorpion antivenom	The immunoglobulin present in antivenom will bind to venom toxins blocking its functional site and thus prevent to show its activity	Birdsall, 2015
Auxiliary treatment	Morphine, Dopamine and dobutamine, Intravenous metoprolol or esmolol and bradyarrhythmiasare, nifedipine, nitroprusside, hydralazine, Captopril, glucoseinsulin-potassium drip, lytic cocktail (pethidine-chlorpromazinepromethazine)	Morphine is used to prevent pain by binding with opioid receptor in central nervous system. Dopamine is used to treat hypotention, bradycardia via its interaction with receptors in pre- and post-synaptic cleft of neurons	Devi et al. (1970); Rahimtoola et al. (1970); Gueron et al. (1992); Sibley et al. (1993); Bawaskar and Bawaskar, (1994); Reddy, (2013); Pandurang et al. (2014); Bhoite et al. (2015); Akella et al. (2016); Junior et al. (2019)

Indian red scorpion venom contains an abundance of low molecular mass toxins, which are poor immunogens (Das et al., 2020), and as a consequence, it can be challenging to raise toxin-specific antibodies in the horse (El Ayeb and Delori, 1984; Kankonkar et al., 1998; Das et al., 2020). Kankonkar et al. (1998) demonstrated that the use of an adjuvant and extending the immunization period resulted in the production of a potent equine antiserum capable of neutralizing the major lethal factors of scorpion venom. However, early administration of antivenom is necessary to prevent the release of catecholamine and minimize the intoxication following scorpion envenomation (Chippaux, 2012).

## Conclusion on Future Directions for Research and Augmentation of Clinical Treatment on Scorpion Sting

Indian red scorpion sting is a typical medical emergency in many Indian sub-tropical countries. Sting severity is more significant in children, the elderly, and immuno-compromised adults and can be lethal if not properly treated. The geographical variation in Indian red scorpion venom toxicity and its pharmacological effects often leads to differences in local and systemic symptoms. In-depth proteomic analyses are necessary to correlate the geographical variation in Indian red scorpion venom composition with sting severity. While various treatments are available for the clinical management of scorpion sting, early administration of scorpion antivenom is the preferred choice, even though the poor immunogenicity of scorpion antivenom might present additional clinical challenges. The adjuvant may be an excellent choice to enhance the antigenicity of low molecular mass toxins of scorpion venom (Kankonkar et al., 1998; Bermúdez-Méndez et al., 2018). Additional studies on scorpion venom composition are warranted to produce highly effective antivenom that will target a broad range of species.

Further, the Indian red scorpion venom is proving to be an essential source of biologically active compounds which could have immense medical and pharmacological value (Strong et al., 2001). Proteomic profiling of the Indian red scorpion venom can provide a catalogue of novel molecules with robust pharmacological characteristics that may be explored as potential life-threatening therapeutics.

While proteomics has identified an abundance of low molecular mass ion channel toxins in Indian red scorpion venom, many proteins or peptides of low abundance may not be identified due to the limited number of sequences in protein reference databases. Therefore, additional –omic analyses, such as genomics and transcriptomics (Zhijian et al., 2006; Ma et al., 2009; Rokyta and Ward, 2017; Ward et al., 2018), are encouraged to promote the discovery of novel scorpion venom toxins. This research will increase our understanding of Indian red scorpion venom (Schendel et al., 2019), its toxicity mechanism, and identify novel drug prototypes from this venom (Uzair et al., 2018).

In addition, venom proteins (toxins) of low abundance can be challenging to detect without optimizing standard mass spectrometric methods or applying alternative techniques, such as western blotting with a particular antibody. Most mass spectrometry-based proteomic experiments are conducted in data-dependent acquisition (DDA) mode, where the stochastic nature of precursor ion selection for tandem MS (MS/MS) creates a bias by omitting low abundance or poorly ionizing peptides (Bateman et al., 2014; Lee et al., 2019). Alternative strategies such as data-independent acquisition (DIA) can enhance the identification and quantification of low abundant proteins (Hu et al., 2016) and could assist in the discovery of novel proteins and peptides from scorpion venoms. Alternatively, sample decomplexing via offline liquid chromatography or gel electrophoresis before mass spectrometry can also promote the identification of low abundant proteins.

Lastly, antibodies raised explicitly against low molecular mass toxins using toxicovenomics, antivenomics, and affinity purification can supplement commercially available scorpion antivenoms and would be an ideal approach for better in-patient management of Indian red scorpion sting. Repurposed drugs can also be explored as possible antidotes to treat scorpion stings.
