# Supplementary material for: Biochemical and Proteomic Characterization, and Pharmacological Insights of Indian Red Scorpion Venom Toxins
Source: Front Pharmacol. 2021 Sep 28;12:710680. doi: 10.3389/fphar.2021.710680 (PMC8505525; doi:10.3389/fphar.2021.710680)
Supplement: Supplementary file 1 [file DataSheet1.docx]

**Supporting information**

| **Sl. No.** | **Name** | **Brief caption** |
| --- | --- | --- |
| **1** | **Supplementary Table S1** | Venom proteome composition of *M. tamulus* deduced by LC-MS/MS analysis. This Supplementary Table was originally published by Das et al., 2020. Published with permission from the publisher. |
| **2** | **Supplementary Table S2** | *M. tamulus* venom toxins with their 3D structures |
| **3** | **Supplementary Table S3** | Distribution of venom toxins from different species of *Mesobuthus and Heterometrus* from the Indian subcontinent |

**Supplementary TableS1.** Proteome composition of *M. tamulus* venom by LC-MS/MS analysis. This Supplementary Table was originally published by Das et al., 2020. Published with permission from the publisher.

| **K channel inhibitor** | | | | | | |
| --- | --- | --- | --- | --- | --- | --- |
| **Accession No.** | **Protein Description** | **Source organism** | **Sequence coverage (%)** | **Identified peptide sequences** | **Morpheus score** | **Theoretical mass (Da)** |
| K7XFK5 | Potassium channel toxin alpha-KTx 3.16 | *Mesobuthus gibbosus* | 48.30% | EIPVKCK | 12 | 872.48 |
|  |  |  |  | VFSAVLIILFVCSMIIGISEGK | 12 | 2395.33 |
|  |  |  |  |  |  |  |
| Q95P89 | Putative potassium channel blocker TXKS1 | *Mesobuthus martensii* | 41.6 | VCKIICGMQGKK | 22 | 1420.74 |
|  |  |  |  | KVNICKAPIK | 8 | 1169.70 |
|  |  |  |  | VAAGIVCKVCKIICGMQGK | 13 | 2107.08 |
|  |  |  |  | MNRLTTIILMLIVINVIMDDISESK | 11 | 2922.55 |
|  |  |  |  | IICGMQGKKVNICK | 9 | 1647.86 |
|  |  |  |  |  |  |  |
| A7KJJ7 | Potassium channel toxin alpha-KTx 26.1 | *Mesobuthus martensii* | 31.50% | VEGACSKPCRK | 11 | 1290.62 |
|  |  |  |  | GARNGKCINGR | 9 | 1201.61 |
|  |  |  |  |  |  |  |
| A9XE59 | Potassium channel toxin MeuTXK-beta-2 | *Mesobuthus eupeus* | 32.90% | FVKYAVPESTLR | 10 | 1408.77 |
|  |  |  |  | TVLQTVVHKVGK | 10 | 1307.79 |
|  |  |  |  | EKHFQR | 7 | 843.44 |
|  |  |  |  |  |  |  |
| B5KF99 | Potassium channel toxin alpha-KTx J123 | *Mesobuthus martensii* | 34.30% | TVCKCSI | 7 | 866.40 |
|  |  |  |  | DMKFGNTGRCTGPNK | 15 | 1697.76 |
|  |  |  |  | FGNTGRCTGPNKTVCK | 12 | 1795.85 |
|  |  |  |  |  |  |  |
| C0HJQ8 | Potassium channel toxin alpha-KTx 1.16 | *Mesobuthus eupeus* | 36.20% | CTGTKQCWPVCK | 8 | 1523.67 |
|  |  |  |  | MFGRPNGKCMNGK | 9 | 1527.67 |
|  |  |  |  | KMFGRPNGK | 9 | 1049.54 |
|  |  |  |  |  |  |  |
| E4VP04 | Potassium channel toxin MeuTXKalpha3 | *Mesobuthus eupeus* | 90.60% | KECRK | 8 | 719.37 |
|  |  |  |  | KLNYR | 6 | 692.40 |
|  |  |  |  | GGKCDR | 6 | 691.31 |
|  |  |  |  | CFPNYCRCFPG | 10 | 1476.57 |
|  |  |  |  | GKCFPNYCR | 8 | 1200.52 |
|  |  |  |  | MKNYCGIITLFLAIISATGVFCVDFPNKGGK | 13 | 3449.75 |
|  |  |  |  |  |  |  |
| Q8I0L5 | Potassium channel toxin alpha-KTx 15.2 | *Mesobuthus martensii* | 50.80% | KAIGVAAGKCINGR | 10 | 1413.79 |
|  |  |  |  | FSSIILLTLLICSMSK | 11 | 1825.01 |
|  |  |  |  |  |  |  |
| Q967F9 | Potassium channel toxin alpha-KTx 14.1 | *Mesobuthus martensii* | 96.20% | CPGNPSCRNGFCACT | 8 | 1756.65 |
|  |  |  |  | IFFAILLILAVCSMAIWTVNGTPFAIKCATDADCSRK | 10 | 4189.12 |
|  |  |  |  |  |  |  |
| Q9NJC6 | Potassium channel toxin BmTXK-beta | *Mesobuthus martensii* | 50% | GKEIMKNIK | 8 | 1075.61 |
|  |  |  |  | AIGKCEDTECK | 8 | 1309.56 |
|  |  |  |  | EKLTEVK | 8 | 845.49 |
|  |  |  |  | EIMKNIKEK | 9 | 1131.63 |
|  |  |  |  | CEDTECKCLK | 8 | 1341.54 |
|  |  |  |  | vi)MMKQQFFLFLAVIVMISSVIEAGRGK | 12 | 2942.59 |
|  |  |  |  | vii)LTEVKDKMK | 7 | 1106.60 |
|  |  |  |  |  |  |  |
| Q86BX0 | Potassium channel toxin alpha-KTx 15.8 | *Mesobuthus martensii* | 96.60% | REIGVAAGKCINGK | 9 | 1471.79 |
|  |  |  |  | FSSIILLTLLICSMSIFGNCQVQTNVKCQGGSCASVCRR | 8 | 4464.17 |
|  |  |  |  | CVCYRN | 6 | 870.35 |
|  |  |  |  |  |  |  |
| C0HJQ2 | Potassium channel toxin alpha-KTx 19.2 | *Buthus occitanus tunetanus* | 54.80% | AMGFSSGKCIDSK | 11 | 1386.63 |
|  |  |  |  | VKCRAMGFSSGK | 9 | 1326.65 |
|  |  |  |  |  |  |  |
| P0DL46 | Potassium channel toxin alpha-KTx 16.9 | *Buthus paris* | 30.50% | GLIDVRCYASR | 7 | 1308.66 |
|  |  |  |  |  |  |  |
| C0HJQ7 | Potassium channel toxin alpha-KTx 1.17 | *Mesobuthus eupeus* | 16.20% | QFTDVK | 6 | 736.38 |
|  |  |  |  |  |  |  |
| Q8MQL0 | Potassium channel toxin alpha-KTx 16.3 | *Mesobuthus martensi* | 20.30% | CFASSECWIACK | 8 | 1517.61 |
|  |  |  |  |  |  |  |
| Q8MUB1 | Potassium channel toxin alpha-KTx 22.1 | *Mesobuthus martensii* | 43.50% | RQNKNGR | 8 | 871.47 |
|  |  |  |  | LFIVFVLFCILRLDAEVDGR | 12 | 2394.31 |
|  |  |  |  |  |  |  |
|  |  |  |  |  |  |  |
| E4VP41 | Potassium channel toxin MeuTXKalpha4 | *Mesobuthus eupeus* | 12.60% | FKACIPYR | 7 | 1053.54 |
|  |  |  |  |  |  |  |
| P0CH57 | Potassium channel toxin MeuTXKbeta3-meucin-24 | *Mesobuthus eupeus* | 26.10% | EFMSNLKEKLSGVK | 12 | 1608.85 |
|  |  |  |  | MKNSWNR | 6 | 934.44 |
|  |  |  |  | LSGVKEKMK | 9 | 1034.58 |
|  |  |  |  |  |  |  |
| Q9BJX2 | Potassium channel toxin alpha-KTx 14.3 | *Mesobuthus martensii* | 46.20% | CPGNPPCRNGFCACT | 10 | 1766.67 |
|  |  |  |  | CATDADCSRK | 9 | 1182.48 |
|  |  |  |  |  |  |  |
| B8XH40 | Potassium channel toxin BuTXK-beta | *Buthus occitanus israeli* | 62.60% | LVKYAVPESTLR | 11 | 1374.79 |
|  |  |  |  | NLVVLLLLGMVALSSCGLREK | 10 | 2300.30 |
|  |  |  |  | KEEGFCHGMKCK | 9 | 1525.65 |
|  |  |  |  | TILQTAVHKLGK | 8 | 1307.79 |
|  |  |  |  |  |  |  |
|  |  |  |  |  |  |  |
| P83112 | Potassium channel toxin alpha-KTx 1.10 | *Parabuthus transvaalicus* | 62% | QATGRPNGKCMNR | 10 | 1504.70 |
|  |  |  |  | EVDMRCKSSK | 9 | 1238.57 |
|  |  |  |  |  |  |  |
| Q9NII5 | Potassium channel toxin alpha-KTx 1.6 | *Mesobuthus martensii* | 91.30% | KLFGTYRGK | 8 | 1068.61 |
|  |  |  |  | ISFLLLLAIVICSIGWTEAQFTNVSCSASSQCWPVCKKLFGTYR | 11 | 5110.56 |
|  |  |  |  | GKCMNSKCR | 8 | 1139.50 |
|  |  |  |  |  |  |  |
|  |  |  |  |  |  |  |
| A0A059UI30 | Potassium channel toxin Meg-beta-KTx1 | *Mesobuthus gibbosus* | 13.10% | KQEGFCHGFKCK | 10 | 1524.70 |
|  |  |  |  | YAVPEGTLR | 10 | 1004.53 |
|  |  |  |  |  |  |  |
|  |  |  |  |  |  |  |
| B8XH44 | Potassium channel toxin alpha-KTx 27.1 | *Buthus occitanus israelis* |  | YGSDCAEPCK | 9 | 1185.44 |
|  |  |  |  | CTCYPSIKIK | 8 | 1268.63 |
|  |  |  |  |  |  |  |
|  |  |  |  |  |  |  |
| C0HJQ4 | Potassium channel toxin alpha-KTx 3.18 | *Mesobuthus eupeus* | 47.50% | EAGMTYGKCMNGKCNCTPK | 13 | 2221.90 |
|  |  |  |  | EIPVKCKGSK | 8 | 1144.63 |
|  |  |  |  |  |  |  |
|  |  |  |  |  |  |  |
| H2ETQ6 | Potassium channel toxin alpha-KTx 1.14 | *Mesobuthus martensii* | 11.80% | KLFGTYK | 7 | 855.49 |
|  |  |  |  |  |  |  |
|  |  |  |  |  |  |  |
| P0DL45 | Potassium channel toxin alpha-KTx 16.8 | *Buthus paris* | 25% | KVTGSGQAK | 9 | 874.49 |
|  |  |  |  |  |  |  |
|  |  |  |  |  |  |  |
| P0DL62 | Potassium channel toxin alpha-KTx 31.1 | *Buthus occitanus tunetanus* | 100% | CPAGECICTT | 7 | 1167.44 |
|  |  |  |  | ACSERIRQVENDNK | 10 | 1717.82 |
|  |  |  |  | AGSMDSCSETGVCMK | 10 | 1618.61 |
|  |  |  |  |  |  |  |
|  |  |  |  |  |  |  |
|  |  |  |  |  |  |  |
| Q9BKB7 | Potassium channel toxin gamma-KTx 2.1 | *Mesobuthus eupeus* | 15.20% | SRFGKTNGR | 8 | 1021.54 |
|  |  |  |  |  |  |  |
|  |  |  |  |  |  |  |
| P0DMR9 | Toxin BmKK16 OS=Mesobuthus martensii | *Mesobuthus martensii* | 34.50% | LRDCYKYCMSPK | 9 | 1635.72 |
|  |  |  |  |  |  |  |
|  |  |  |  |  |  |  |
| B3EWY1 | Potassium channel toxin alpha-KTx 16.7 | *Mesobuthus gibbosus* | 25.40% | KVTGSGQGKCQNNQCR | 10 | 1820.84 |
|  |  |  |  |  |  |  |
|  |  |  |  |  |  |  |
| P46114 | Potassium channel toxin alpha-KTx 4.1 | *Tityus serrulatus* | 24.30% | EAIGKAAGK | 9 | 843.48 |
|  |  |  |  |  |  |  |
|  |  |  |  |  |  |  |
| Q5F1N4 | Toxin BmTxKS4 | *Mesobuthus martensii* | 28.20% | MLCGIDGKLRESK | 10 | 1521.76 |
| Q5F1N4 | Toxin BmTxKS4 | *Mesobuthus martensii* |  | WFPASVNGK | 8 | 1004.51 |
|  |  |  |  |  |  |  |
| B8XH30 | Potassium channel toxin alpha-KTx Tx308 | *Buthus occitanus israelis* | 53.20% | LFIVLLLFCILRLDAEVDGRTMSHCNQSECQEK | 11 | 4009.95 |
|  |  |  |  |  |  |  |
| B8XH38 | Potassium channel toxin-like Tx677 | *Buthus occitanus israelis* | 13.30% | AAKCINRK | 7 | 959.53 |
|  |  |  |  |  |  |  |
|  |  |  |  |  |  |  |
| B8XH45 | Potassium channel toxin alpha-KTx Tx773 | *Buthus occitanus israelis* | 59.60% | LDAEVDGRR | 8 | 1029.52 |
|  |  |  |  | KENKNGR | 10 | 844.45 |
|  |  |  |  | QPGCQEACKKENK | 12 | 1575.71 |
|  |  |  |  | LFIVLLLFCILR | 9 | 1518.94 |
|  |  |  |  |  |  |  |
| Q9NJP7 | Potassium channel toxin alpha-KTx 9.1 | *Mesobuthus martensii* | 53.50% | LFTLVLIVLAMNVMMAIISDPVVEAVGCEECPMHCKGK | 10 | 4322.10 |
|  |  |  |  |  |  |  |
| P0DL65 | Mesomartoxin | *Mesobuthus martensii* | 32.20% | YCQDKGARNGK | 11 | 1295.60 |
|  |  |  |  | VEGACVENCR | 11 | 1192.50 |
|  |  |  |  | GARNGKCINSNCHCYY | 11 | 1972.81 |
|  |  |  |  | KYCQDKGAR | 9 | 1124.54 |
|  |  |  |  |  |  |  |
| B8XH42 | Potassium channel toxin alpha-KTx 16.6 | *Buthus occitanus israelis* | 15.50% | RVTGSAQAK | 9 | 916.51 |
|  |  |  |  |  |  |  |
| P0DMR8 | Toxin BmKK12 | *Mesobuthus martensii* |  | QCQNVQNCYKYCMSPK | 9 | 2122.87 |
|  |  |  |  |  |  |  |
| A0ASK0 | Potassium channel toxin alpha-KTx 14.x | *Mesobuthus martensii* | 70.30% | MKIFFAILLILAVCSMAIWTVNGTPFEVRCATDADCAR | 10 | 4360.17 |
|  |  |  |  |  |  |  |
| C0HJQ5 | Potassium channel toxin alpha-KTx 8.7 | *Mesobuthus eupeus* | 17.50% | DQRAKCENDK | 8 | 1262.57 |
|  |  |  |  |  |  |  |
| P86400 | Potassium channel toxin alpha-KTx 8.6 | *Mesobuthus eupeus* | 43.80% | MSRLYAIILIALVFNVIMTIMPDMK | 11 | 2943.57 |
|  |  |  |  |  |  |  |
| \|Q9BKB4 | Potassium channel toxin alpha-KTx 14.4 | *Mesobuthus martensii* | 16.60% | CATNADCSR | 8 | 1053.40 |
|  |  |  |  |  |  |  |
| P83407 | Potassium channel toxin alpha-KTx 19.1 | *Mesobuthus martensii* | 48.30% | CVAMGFSSGKCINSK | 14 | 1644.74 |
|  |  |  |  |  |  |  |
| P86402 | Neurotoxin MeuClTx-1 | *Mesobuthus eupeus* | 71.40% | MCMPCFTTRPDMAQQCRDCCGGNGK | 11 | 3053.16 |
|  |  |  |  |  |  |  |

| **Na^+^ channel inhibitor** | | | | | | |  |
| --- | --- | --- | --- | --- | --- | --- | --- |
| **Accession No.** | **Protein Description** | **Source organism** | **Sequence coverage (%)** | **Base Peptide Sequence** | **Morpheus Score** | **Theoretical mass(Da)** | |
| P01485 | Alpha-mammal toxin Bot3 | *Buthus occitanus tunetanus* | 52.70% | LVMAGVESVKDGYIVDDRNCTYFCGR | 40 | 3023.39 | |
|  |  |  |  | VPDHVRTKGPGR | 11 | 1317.73 | |
|  |  |  |  |  |  |  | |
| M4GX67 | BmKBT-like peptide | *Mesobuthus martensii* | 63.80% | KSGYPIQHDGCK | 12 | 1388.65 | |
|  |  |  |  | MKAALLLVISTLMLIGVLTKK | 14 | 2255.41 | |
|  |  |  |  | LACWCDNIHNWVPTWSRETNK | 13 | 2686.22 | |
|  |  |  |  |  |  |  | |
| P0CF76 | Toxin BmKNJX11 | *Mesobuthus martensii* | 86.60% | DAYIADSENCTYT | 8 | 1521.59 | |
|  |  |  |  |  |  |  | |
| Q4TUA4 | Alpha-toxin 4 | *Mesobuthus martensii* | 43.50% | VPIRVPGRCNGG | 8 | 1280.68 | |
|  |  |  |  | LPDKVPIRVPGR | 11 | 1345.82 | |
|  |  |  |  |  |  |  | |
| Q9GNG8 | Toxin BmKaTX15 | *Mesobuthus martensii* | 72.90% | NGAESGYCQWAGVYGNACWCYKLPDK | 11 | 3053.29 | |
|  |  |  |  | VPIRVPGKCNGG | 9 | 1252.67 | |
|  |  |  |  | MNYLVFFSLALLVMTGVESVRDGYIADDK | 11 | 3297.63 | |
|  |  |  |  |  |  |  | |
| Q9GQV6 | Toxin BmKaTx16 | *Mesobuthus martensii* | 15.40% | ELPDNVPIRVPGK | 11 | 1432.80 | |
|  |  |  |  |  |  |  | |
| Q9NBW2 | Toxin BmKBT | *Mesobuthus martensii* | 54.20% | MKAALLLVIFSLMLIGVLTK | 10 | 2173.33 | |
|  |  |  |  | LACWCDDIHNWVPTWSRATNKCR | 12 | 2945.33 | |
|  |  |  |  | ATNKCRAK | 8 | 947.50 | |
|  |  |  |  |  |  |  | |
| Q9NJC4 | Toxin BmKaTx17 | *Mesobuthus martensi* | 54% | LLMTGVESGRDAYIAK | 9 | 1722.90 | |
|  |  |  |  | NYNCVYHCFR | 8 | 1431.58 | |
|  |  |  |  | YGNACWCINLPDDK | 9 | 1724.73 | |
|  |  |  |  |  |  |  | |
|  |  |  |  |  |  |  | |
| Q9NJC5 | BmKaTx10 | *Mesobuthus martensii* | 35.20% | IGYCNIQGK | 9 | 1051.51 | |
|  |  |  |  |  |  |  | |
| Q9NJC7 | BmK AGP-SYPU2 | *Mesobuthus martensii* | 89.40% | NRAESGYCQWASK | 9 | 1555.68 | |
|  |  |  |  | PGRCNGG | 6 | 716.30 | |
|  |  |  |  | NAYCDGECKK | 10 | 1243.50 | |
|  |  |  |  | MNYMVIISLALLVMTGVESVKDGYIADDR | 10 | 3247.62 | |
|  |  |  |  | IMKPGRCNGG | 8 | 1104.52 | |
|  |  |  |  | DGYIADDRNCPYFCGR | 12 | 1977.81 | |
|  |  |  |  | KNRAESGYCQWASK | 11 | 1683.78 | |
|  |  |  |  | LPDDARIMKPGR | 9 | 1383.73 | |
|  |  |  |  |  |  |  | |
| Q9UAC8 | Beta-toxin BmKAs1 | *Mesobuthus martensii* | 40% | LACYCEGAPKSELWAYETNK | 11 | 2389.07 | |
|  |  |  |  | ADNGYLLNK | 9 | 1006.51 | |
|  |  |  |  | CNGKM | 6 | 624.24 | |
|  |  |  |  | SELWAYETNKCNGK | 11 | 1698.77 | |
|  |  |  |  |  |  |  | |
| P0C5F0 | Alpha-toxin PgKL1 | *Parabuthus granulatu* | 22.50% | IDGYPVDNWNCKR | 9 | 1635.75 | |
|  |  |  |  | KIDGYPVDNWNCK | 9 | 1607.74 | |
|  |  |  |  |  |  |  | |
| P01486 | Alpha-toxin Bot11 | *Buthus occitanus tunetanus* | 49.20% | LKDGYIVDDR | 9 | 1192.61 | |
|  |  |  |  | YGNACWCYK | 9 | 1220.47 | |
|  |  |  |  | LKGESGYCQWVGR | 9 | 1538.73 | |
|  |  |  |  | LPDHVRTVQAGRCR | 8 | 1663.87 | |
|  |  |  |  |  |  |  | |
| Q9GYX2 | Toxin BmKa1 | *Mesobuthus martensii* | 43.50% | NYLVFFSLALLLMTGVGSVRDGYIADDKNCPYFCGR | 9 | 4147.00 | |
|  |  |  |  |  |  |  | |
| P01488 | Alpha-toxin Bot1 | *Buthus occitanus*  *tunetanus* | 40% | NGATSGYCQWLGK | 10 | 1440.65 | |
|  |  |  |  | DLPDNVPIRIPGK | 9 | 1432.80 | |
| P58328 | Alpha-like toxin BmK-M4 | *Mesobuthus martensii* | 28.10% | LPDDVPIRVPGKCH | 10 | 1601.83 | |
|  |  |  |  |  |  |  | |
| P59354 | Alpha-like toxin Bom4 | *Buthus occitanus*  *mardochei* | 20% | NGAKSGYCQWLGK | 9 | 1467.69 | |
|  |  |  |  |  |  |  | |
| P60256 | Toxin Boma6b | *Buthus occitanus*  *mardochei* | 12.10% | VEGKCHRK | 9 | 1012.52 | |
|  |  |  |  |  |  |  | |
| P82815 | Bukatoxin | *Mesobuthus martensii* | 50.70% | LPDKVPIRVSGECQQ | 9 | 1724.89 | |
|  |  |  |  | VRDGYIADDK | 9 | 1150.56 | |
|  |  |  |  | NCAYFCGR | 8 | 1046.41 | |
|  |  |  |  |  |  |  | |
| Q9GQW3 | Toxin BmKaIT1 | *Mesobuthus martensii* | 42.30% | NYLVMISFAFLLMTGVESVRDAYIAQNYNCVYHCAR | 8 | 4320.03 | |
|  |  |  |  | NGAKSGSCPYLGEHK | 11 | 1603.74 | |
|  |  |  |  |  |  |  | |
| P01490 | Alpha-toxin BeM10 | *Mesobuthus eupeus* | 69.20% | NAYCDEECKKGAESGK | 9 | 1844.77 | |
|  |  |  |  | LPDWVPIKQKVSGK | 8 | 1593.92 | |
|  |  |  |  | GAESGKCWYAGQYGNACWCYK | 13 | 2515.01 | |
|  |  |  |  |  |  |  | |
| P09982 | Toxin BeM14 | *Mesobuthus eupeus* | 28.70% | NLPDDVPIR | 9 | 1037.55 | |
|  |  |  |  | ARDAYIADDR | 9 | 1164.55 | |
|  |  |  |  |  |  |  | |
| P54135 | Alpha-mammal toxin BmK-M8 | *Mesobuthus martensii* | 23.40% | IKEPGKCG | 8 | 887.45 | |
|  |  |  |  |  |  |  | |
| P58488 | Alpha-like toxin BmK-M2 | *Mesobuthus martensii* | 14% | SGYCQWSGK | 9 | 1071.44 | |
|  |  |  |  |  |  |  | |
| Q17231 | Toxin BmKIT3 | *Mesobuthus martensii* | 31.20% | SESNTCGRKK | 7 | 1165.55 | |
|  |  |  |  | DGYIRGSNGCK | 12 | 1225.55 | |
|  |  |  |  |  |  |  | |
| P58910 | Kurtoxin | *Parabuthus transvaalicus* | 22.50% | KIDGYPVDYWNCKR | 12 | 1812.86 | |
|  |  |  |  |  |  |  | |
| Q9GUA7 | Toxin BmKa3 | *Mesobuthus martensii* | 34.10% | GAESGYCQWAGVYGNACWCYKLPDKVPIR | 9 | 3404.55 | |
|  |  |  |  |  |  |  | |
| P13488 | Alpha-like toxin Bom3 | *Buthus occitanus*  *mardochei* | 16.60% | VPIVVGGEKCH | 8 | 1193.62 | |
|  |  |  |  |  |  |  | |
| P59854 | Alpha-like toxin BmK-M7 | *Mesobuthus martensii* | 12.10% | VPGRCHPA | 9 | 892.43 | |
|  |  |  |  |  |  |  | |
| Q8I0K7 | Depressant scorpion toxin BmKIM | *Mesobuthus martensii* | 29.40% | ISCLWGNEGCNKECK | 14 | 1853.79 | |
|  |  |  |  | SESNTCGGKK | 12 | 1066.47 | |
|  |  |  |  |  |  |  | |
| P0DMH9 | Alpha-toxin BmalphaTx47 | *Mesobuthus martensii* | 9.40% | ISGSCRGR | 7 | 891.43 | |
|  |  |  |  |  |  |  | |
| E7CAU3 | Neurotoxin BmK AGP-SYPU1 | *Mesobuthus martensii* | 21.20% | YGHACWCINLPDDK | 9 | 1747.74 | |
|  |  |  |  |  |  |  | |
| P01483 | Neurotoxin Bot2 | *Buthus occitanus*  *tunetanus* | 24.60% | SGYCQWLGR | 8 | 1125.50 | |
|  |  |  |  | IEGKCHF | 7 | 889.41 | |
|  |  |  |  |  |  |  | |
| G4V3T9 | Neurotoxin BmK AGAP-SYPU2 | *Mesobuthus martensii* | 26.10% | VKDGYIVDDK | 8 | 1150.59 | |
|  |  |  |  | VPGRCNG | 8 | 758.35 | |
|  |  |  |  |  |  |  | |
| P86408 | Neurotoxin MeuNaTx-1 | *Mesobuthus eupeus* | 49.40% | NCAYFCGRNAYCDEECK | 13 | 2215.82 | |
|  |  |  |  | GAESGYCQWAGQYGNACWCYKLPDK | 12 | 2968.24 | |
|  |  |  |  |  |  |  | |
| P86404 | Neurotoxin MeuNaTx-4 | *Mesobuthus eupeus* | 6.90% | NGAKSGYCQILGIYGNGCWCIALPDNVPIR | 43 | 3365.61 | |
|  |  |  |  | IPGKCH | 6 | 710.35 | |
|  |  |  |  |  |  |  | |
| Q9N682 | A Neurotoxin BmK-M11 | *Mesobuthus martensii* | 46.40% | DAYIAKPENCVYHCATNEGCNKLCTDNGAESGYCQWGGK | 42 | 4496.86 | |
|  |  |  |  |  |  |  | |
| O61705 | Neurotoxin BmK-M10 | *Mesobuthus martensii* | 35.70% | VPGKCQR | 7 | 843.44 | |
|  |  |  |  | LPDSVPIRVPGK | 13 | 1276.75 | |
|  |  |  |  | NYLVMISFALLLMK | 8 | 1686.91 | |
|  |  |  |  |  |  |  | |
| P45698 | Neurotoxin BmK-M9 | *Mesobuthus martensii* | 36.70% | MISFALLLMTGVESVR | 12 | 1781.94 | |
|  |  |  |  |  |  |  | |
| P86403 | Neurotoxin MeuNaTx-2 | *Mesobuthus eupeus* | 68.20% | FGNACWCKNLPDK | 10 | 1608.72 | |
|  |  |  |  | NLPDKVPIR | 14 | 1050.62 | |
|  |  |  |  | KNGADSGYCQWFGRFGNACWCK | 12 | 2668.12 | |
|  |  |  |  | ARDAYIANDRNCVYTCALNPYCDSECK | 12 | 3298.39 | |
|  |  |  |  |  |  |  | |
| P86406 | Neurotoxin MeuNaTx-6 | *Mesobuthus eupeus* | 29% | LACYCEGAPKSELWHYETNKCNGR | 12 | 2942.29 | |

| **Serine protease- like protein** | | | | | | |
| --- | --- | --- | --- | --- | --- | --- |
| **Accession No.** | **Protein Description** | **Source organism** | **Sequence coverage (%)** | **Base Peptide Sequence** | **Morpheus Score** | **Theoretical Mass (Da)** |
| P0C8M2 | Serine proteinase-like BMK-CBP | *Mesobuthus martensii* | 40% | IFGGTFAK | 14.01938846 | 839.45 |
|  |  |  |  | KFVLTAAH | 10.00816679 | 885.50 |

| **Serine protease inhibitor** | | | | | | |
| --- | --- | --- | --- | --- | --- | --- |
| **Accession No.** | **Protein Description** | **Source organism** | **Sequence coverage(%)** | **Base Peptide Sequence** | **Morpheus Score** | **Theoretical Mass(Da)** |
| P0DJ47 | Kunitz-type serine protease inhibitor BmKTT-3 | *Mesobuthus martensii* | 60% | KRHGWLGTGWI | 9 | 1309.70 |
|  |  |  |  | HGSINCRLPPER | 9 | 1434.72 |
|  |  |  |  | NRHYCMKYCAR | 8 | 1557.68 |
|  |  |  |  | KHGSINCRLPPER | 8 | 1562.81 |
|  |  |  |  | YYYHNESR | 7 | 1130.48 |
|  |  |  |  |  |  |  |
| P0DJ49 | Kunitz-type serine protease inhibitor BmKTT-1 | *Mesobuthus martensii* | 64.40% | TCESFIYGGVGGNK | 10 | 1487.67 |
|  |  |  |  | QKDCSLPVDTGR | 9 | 1374.66 |
|  |  |  |  | GKGWFLRYYYNK | 8 | 1593.81 |

| **Parabutoporin** | | | | | | |
| --- | --- | --- | --- | --- | --- | --- |
| **Accession No.** | **Protein Description** | **Source organism** | **Sequence coverage(%)** | **Base Peptide Sequence** | **Morpheus Score** | **Theoretical Mass (Da)** |
| P83312 | Parabutoporin | *Parabuthus schlechteri* | 91.1% | SKLAKK | 7 | 673.45 |
|  |  |  |  | FKLGSFLKK | 8 | 1066.65 |
|  |  |  |  | LGSFLKKAWK | 8 | 1176.70 |
|  |  |  |  | GKEMLKDYAK | 9 | 1181.61 |
|  |  |  |  | GLLEGGSEEVPGQ | 8 | 1270.60 |

| **Lipolysis-potentiating peptides.** | | | | | | |
| --- | --- | --- | --- | --- | --- | --- |
| **Accession No.** | **Protein Description** | **Source organism** | **Sequence coverage(%)** | **Base Peptide Sequence** | **Morpheus Score** | **Theoretical Mass (Da)** |
| P84809 | Lipolysis-activating peptide 1-beta chain | *Buthus occitanus tunetanus* | 69.4% | VCKMHLARGGGR | 22 | 1356.69 |
|  |  |  |  | QCPLLKG | 7 | 814.44 |
|  |  |  |  | MISVQVIFIAFISIIAFSMVCGGNVFPNRELGILYGCK | 12 | 4279.21 |
|  |  |  |  | GYGNAFCDK | 7 | 1030.42 |
|  |  |  |  |  |  |  |
| B8XGZ8 | Lipolysis-activating peptide 1-beta chain | *Buthus occitanus israelis* | 52% | ICKLHLAKK | 16 | 1109.67 |
|  |  |  |  | GGFCHQPAPFVELCKCLDIDYDNTYFLKAMEK | 10 | 3865.76 |
|  |  |  |  | AMEKQCPK | 8 | 990.46 |
|  |  |  |  | QCPKLKGNVN | 9 | 1156.60 |
|  |  |  |  |  |  |  |
| Q6WJF5 | Lipolysis-activating peptide 1-alpha chain | *Mesobuthus martensii* | 45.9% | CWCEKLEDK | 8 | 1266.54 |
|  |  |  |  | MKFVLFGMIVILFSLMGSIRGDDDPGNYPTNAYGNK | 12 | 3995.96 |

| **Bradykinin-potentiating peptide** | | | | | | |
| --- | --- | --- | --- | --- | --- | --- |
| **Accession No.** | **Protein Description** | **Source organism** | **Sequence coverage(%)** | **Base Peptide Sequence** | **Morpheus Score** | **Theoretical Mass (Da)** |
| Q9TWD3 | Bradykinin-potentiating peptide K12 | *Buthus occitanus* | 100% | LRDYANRVINGGPVEAAGPPA | 10 | 2136.11 |
|  |  |  |  |  |  |  |
| Q9Y0X4 | Bradykinin-potentiating peptide BmKbpp | *Mesobuthus martensii* | 15.2% | GKQLLKDYANK | 8 | 1276.71 |

| **Antimicrobial peptide** | | | | | | |
| --- | --- | --- | --- | --- | --- | --- |
| **Accession No.** | **Protein Description** | **Source organism** | **Sequence coverage(%)** | **Base Peptide Sequence** | **Morpheus Score** | **Theoretical Mass (Da)** |
| Q6JQN2 | Peptide BmKn2 | *Mesobuthus martensii* | 8.5% | YLYDPSLSAADLK | 23 | 1454.73 |
|  |  |  |  | DMDTMK | 7 | 755.28 |
|  |  |  |  |  |  |  |
| B8XH50 | Amphipathic peptide Tx348 | *Buthus occitanus israelis* | 14.9% | RSMRNMDTMK | 9 | 1300.57 |
|  |  |  |  |  |  |  |
| Q9GQW4 | Peptide BmKn1 | *Mesobuthus martensii* | 62.8% | MKSQTFFLLFLVVLLLAISQSEAFIGAVAGLLSKIFGK | 9 | 4099.36 |
|  |  |  |  |  |  |  |
| E4VP07 | Venom antimicrobial peptide-6 | *Mesobuthus eupeus* | 68.5% | SLRDMDTMK | 10 | 1111.50 |
|  |  |  |  | MKSQTFFLLFLVVFLLAITQSEAIFGAIAGLLKNIFGK | 9 | 4188.38 |
|  |  |  |  | NIFGKRSLR | 7 | 1089.64 |

| **Hyaluronidase** |  |  |  |  |  |  |
| --- | --- | --- | --- | --- | --- | --- |
| **Accession No.** | **Protein Description** | **Source organism** | **Sequence coverage(%)** | **Base Peptide Sequence** | **Morpheus Score** | **Theoretical Mass (Da)** |
|  |  |  |  |  |  |  |
| P86100 | Hyaluronidase-1 | *Mesobuthus martensii* | 54% | HTNISCKCK | 8 | 1146.53 |
|  |  |  |  | NPTFKHTNISCK | 8 | 1445.71 |
|  |  |  |  | KTVPSMDFKR | 9 | 1223.63 |
|  |  |  |  | GNCVWPEEPYTSWK | 9 | 1751.76 |
|  |  |  |  | GGYTGR | 6 | 609.29 |
|  |  |  |  | VAIEEWENSAKEWMLK | 11 | 1977.95 |
|  |  |  |  | EMKTYVK | 7 | 897.46 |
|  |  |  |  | YNTSQR | 6 | 767.36 |
|  |  |  |  | VVWEVPSIMCSKK | 11 | 1561.80 |
|  |  |  |  | ILVNQEETFNGDK | 9 | 1505.74 |
|  |  |  |  | SKDLVKAK | 9 | 887.54 |
|  |  |  |  | IARDNISK | 8 | 915.51 |
|  |  |  |  | INVTDLLTSHK | 11 | 1239.68 |
|  |  |  |  | YLIDPKNPTFK | 9 | 1334.72 |
|  |  |  |  | AKHPDWSPAQIEK | 9 | 1505.76 |
|  |  |  |  | DQPSEYFCKNDIQEANDK | 13 | 2199.94 |
|  |  |  |  | ETIRLSHPNTLIYPYINYILPGTKK | 12 | 2943.62 |
|  |  |  |  | IVIFYESQLGKYPHIESHGDINGGMLQVSDLANHLK | 12 | 4038.03 |

| **Cl^-^ channel inhibitor** | | | | | | |
| --- | --- | --- | --- | --- | --- | --- |
| **Accession No.** | **Protein Description** | **Source organism** | **Sequence coverage (%)** | **Base Peptide Sequence** | **Morpheus Score** | **Theoretical Mass (Da)** |
| Q9BJW4 | Neurotoxin Bm12-b | *Mesobuthus martensii* | 77.9% | ECCGGNGK | 9 | 880.32 |
|  |  |  |  |  |  |  |
| Q9UAD0 | Neurotoxin BmK CT | *Mesobuthus martensii* | 81.35% | KCRECCGGIGK | 8 | 1323.58 |
|  |  |  |  | MKFLYGIVFIALFLTVMFATQTDGCGPCFTTDANMARK | 9 | 4351.10 |

| **Ca^2+^ channelinhibitor** | | | | | | |
| --- | --- | --- | --- | --- | --- | --- |
| **Accession No.** | **Protein Description** | **Source organism** | **Sequence coverage (%)** | **Base Peptide Sequence** | **Morpheus Score** | **Theoretical Mass (Da)** |
| Q8I6X9 | Toxin BmCa-1 | *Mesobuthus martensii* | 31.25% | GCNRLNK | 8 | 860.43 |
|  |  |  |  | CNSDGDCCRYGER | 8 | 1647.58 |

| **HMG-CoA reductase inhibitor** | | | | | | |
| --- | --- | --- | --- | --- | --- | --- |
| **Accession No.** | **Protein Description** | **Source organism** | **Sequence coverage(%)** | **Base Peptide Sequence** | **Morpheus Score** | **Theoretical Mass (Da)** |
| Q95P90 | HMG-CoA reductase inhibitor bumarsin | *Mesobuthus martensii* | 18% | LHLASGGSCQQPAPFVK | 12 | 1795.90 |

| **Proteins with unknown target** | | | | | | |
| --- | --- | --- | --- | --- | --- | --- |
| **Accession No.** | **Protein Description** | **Source organism** | **Sequence coverage(%)** | **Base Peptide Sequence** | **Morpheus Score** | **Theoretical Mass (Da)** |
| P0CH58 | Meucin-25 | *Mesobuthus eupeus* | 62.50% | IEYSLVQLLLRNVTIPLLLIIQMHIMSSVKLIQIR | 8 | 4100.43 |
| Q9Y0X6 | BmK-YA precursor | *Mesobuthus martensii* | 13.5% | SDEERQDWIPSDYGGHMNPAGRSNEER | 13 | 3147.33 |
| P0C5J8 | Toxin Plt | *Parabuthus liosoma* | 52.9% | FKVQR | 7 | 676.40 |
|  |  |  |  | LCEKFK | 6 | 823.43 |
| Q7M463 | Neurotoxin BmK A3-6 | *Mesobuthus martensii* | 72.4% | TECECVMCGLGIICKQCYYQQ | 9 | 2699.10 |
|  |  |  |  |  |  |  |
| Q7Z0H4 | Neurotoxin BmP08 | *Mesobuthus martensii* | 16.3% | NGYCQGCTR | 7 | 1114.43 |
| P15220 | Insectotoxin-I1 | *Mesobuthus eupeus* | 58.3% | MCMPCFTTRPDMAQQCRACCK | 9 | 2724.06 |
| P15222 | Insectotoxin-I5A | *Mesobuthus eupeus* | 22% | DCCGGNGK | 7 | 866.30 |
|  |  |  |  | CMPCFTTDPNMAKK | 9 | 1699.72 |
|  |  |  |  |  |  |  |
| P60270 | Insectotoxin-I5 | *Mesobuthus eupeus* | 51.4% | DCCGGGKKCFGPQCLCNR | 12 | 2172.87 |
| P60268 | Insectotoxin-I3 | *Mesobuthus eupeus* | 25% | RCRDCCGGR | 8 | 1195.48 |
| D2CFI7 | Venom peptide MmKTx1 | *Mesobuthus martensii* | 40.6% | SLRRYYFSK | 8 | 1218.65 |
|  |  |  |  | TCATVFYPSNCR | 12 | 1474.63 |
|  |  |  |  | IDTCKTLTGETIK | 10 | 1478.76 |
|  |  |  |  | EEPGTGLYPDCCNK | 8 | 1638.66 |
|  |  |  |  | GRIDTCK | 7 | 848.41 |

| **Makatoxin** |  |  |  |  |  |  |
| --- | --- | --- | --- | --- | --- | --- |
| **Accession No.**  Q86BW9 | **Protein Description**  Makatoxin-2 | **Source organism**  *Mesobuthus martensii* | **Sequence coverage(%)**  27% | **Base Peptide Sequence** | **Morpheus Score** | **Theoretical Mass (Da)** |
|  |  |  |  | YGNACWCIDLPDK | 9 | 1610.69 |
|  |  |  |  | VPIRIPGPCR | 7 | 1163.66 |
| P59853 | Makatoxin-3 | *Mesobuthus martensii* | 52.9% | YGNACWCIDLPDK | 9 | 1610.69 |
|  |  |  |  | NYLIVISFALLLMTGVESGR | 12 | 2211.20 |
|  |  |  |  | VPIRIPGPCIGR | 9 | 1333.77 |

| **Supplementary table1:** The three-dimensional (3D) structures*M. tamulus* venom toxins. | | | |
| --- | --- | --- | --- |
| **Sl. No.** | **Toxins** | **Accession number** | **3D structure** |
| 1 | Na^+^ channel toxin (α neurotoxin) | Q9NJC4 | 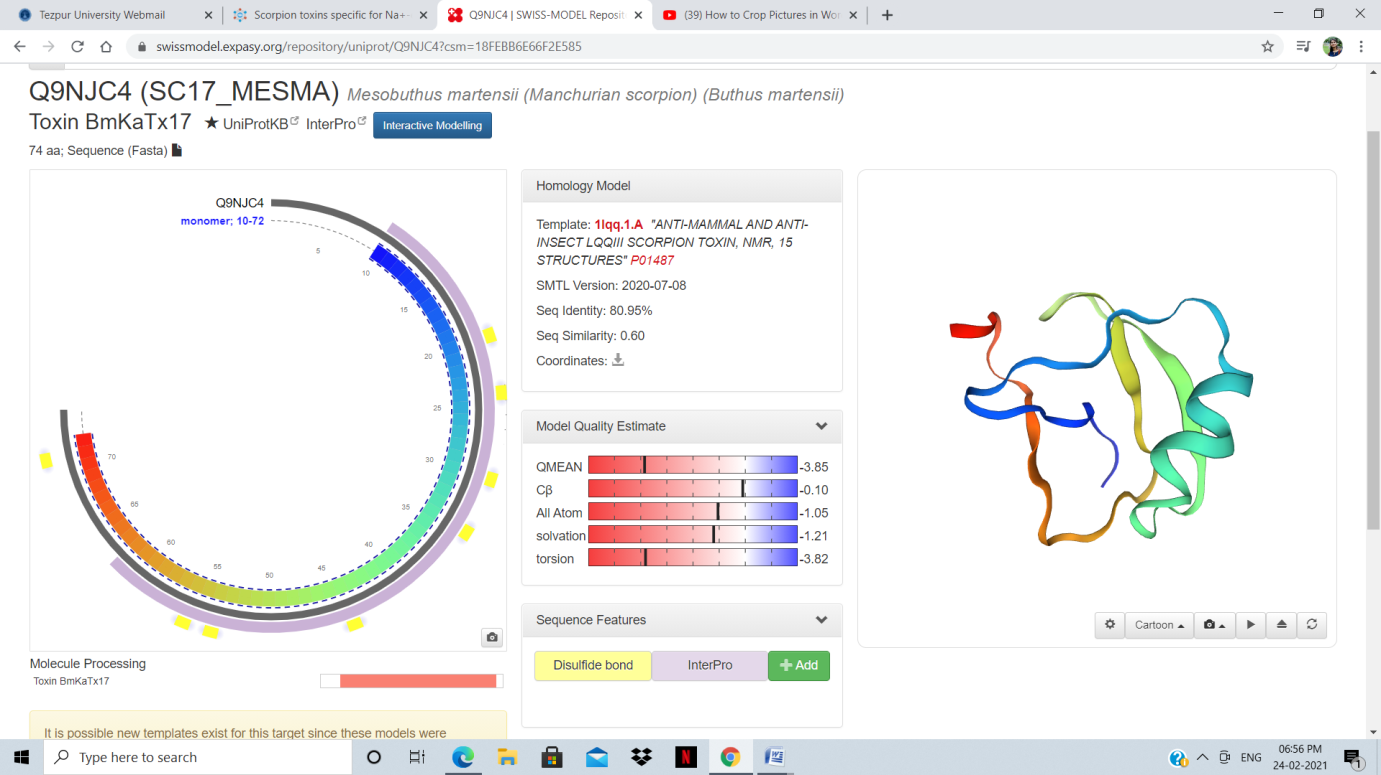 |
| 2 | Na^+^ channel toxin (β neurotoxin) | Q9UAC8 | 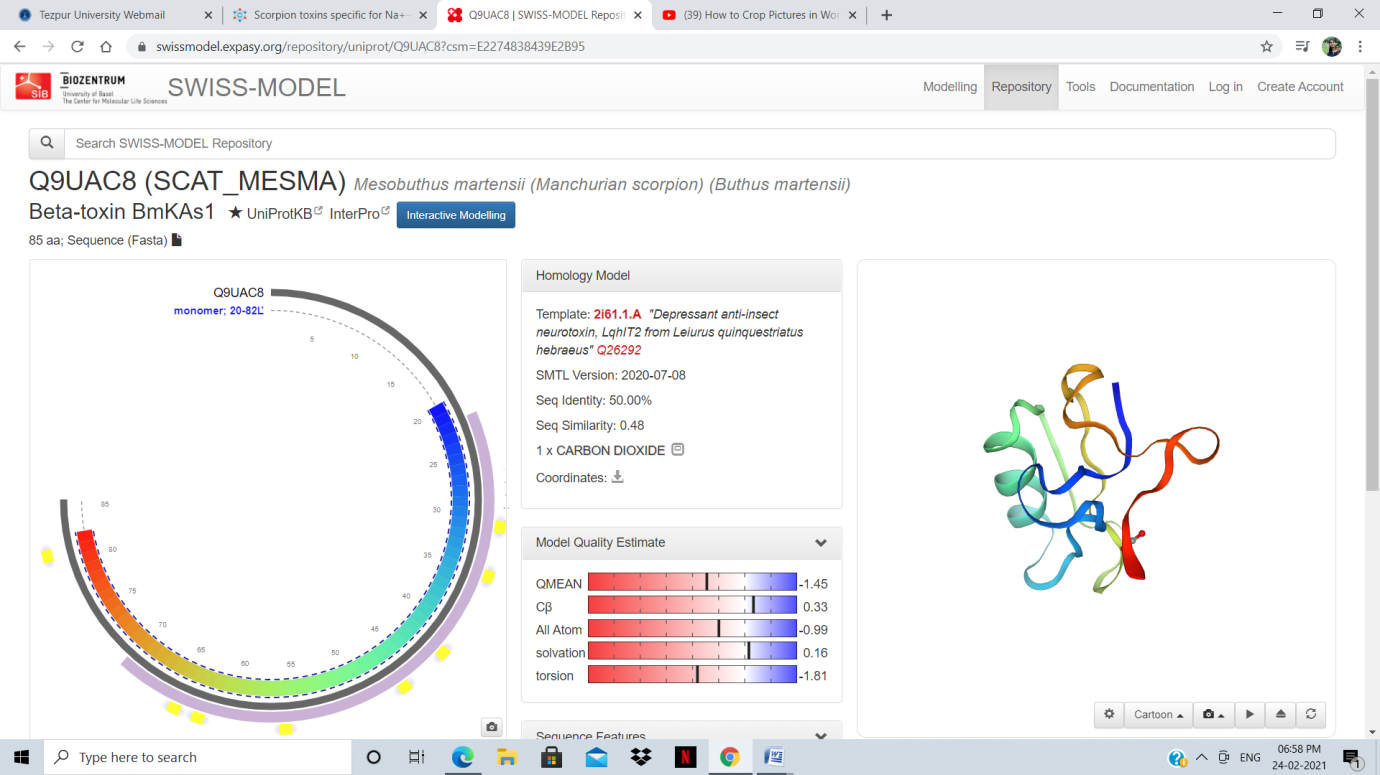 |
| 3 | K^+^ channel toxin | K7XFK5 | 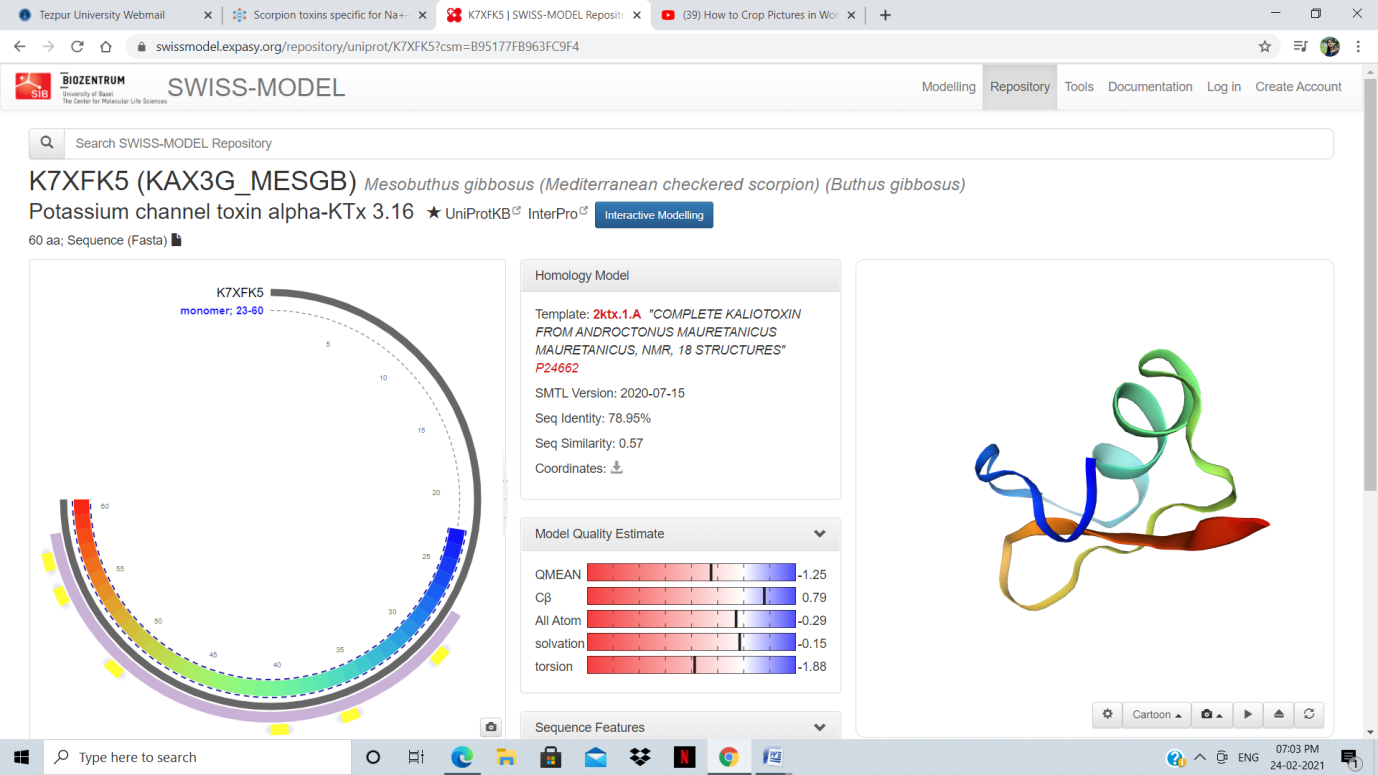 |
| 4 | Cl^-^ channel toxin | Q9BJW4 | 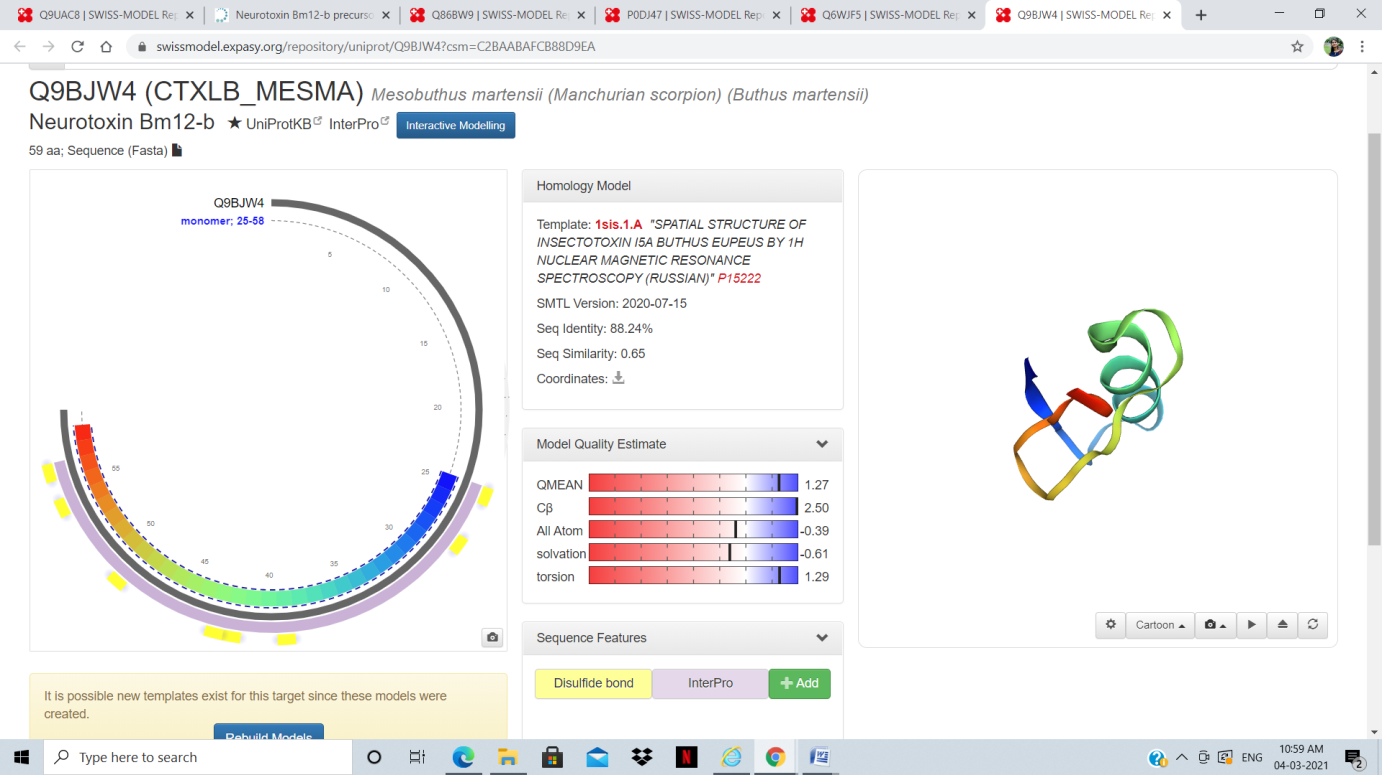 |
| 5 | Ca^2+^ channel toxin | Q8I6X9 | 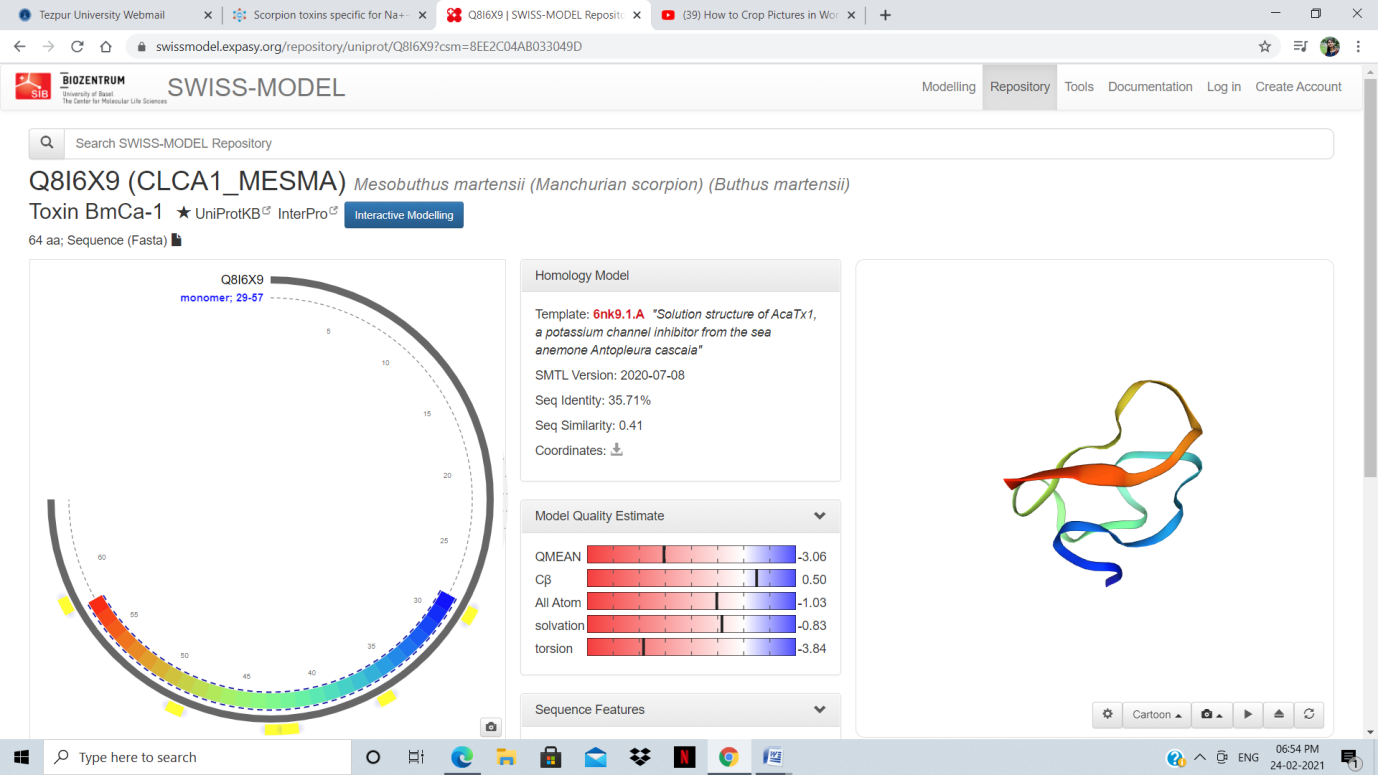 |
| 6 | Hyaluronidase | P86100 | 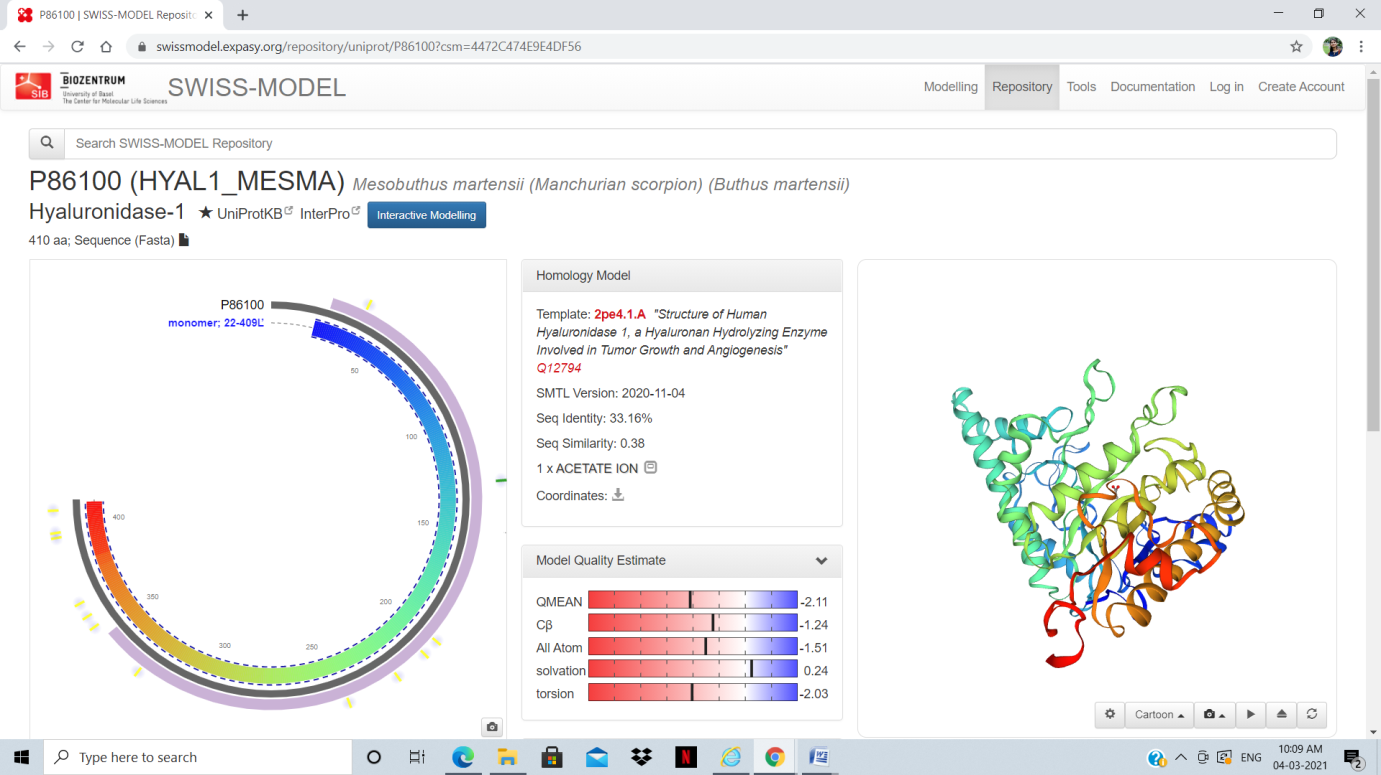 |
| 7 | Bukatoxin | P82815 | 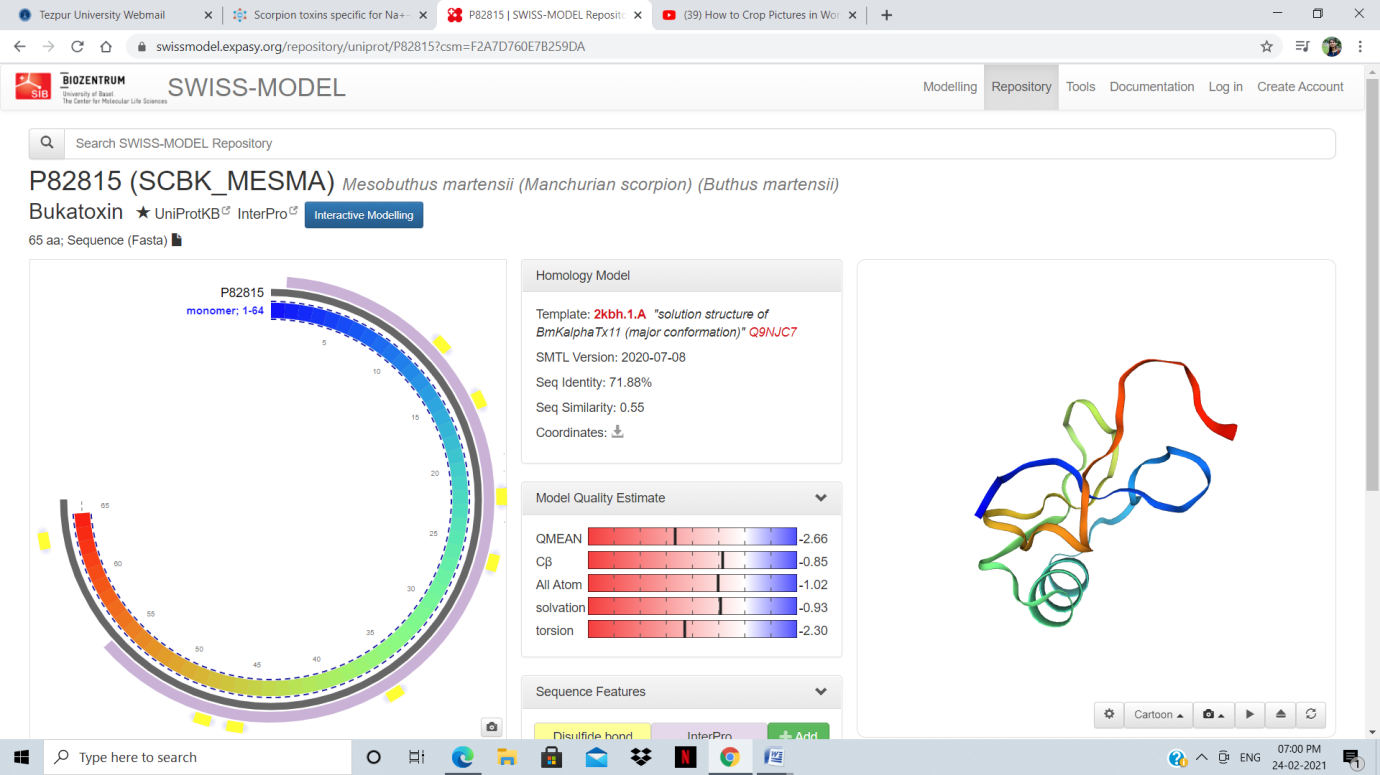 |
| 8 | Makatoxin | Q86BW9 | 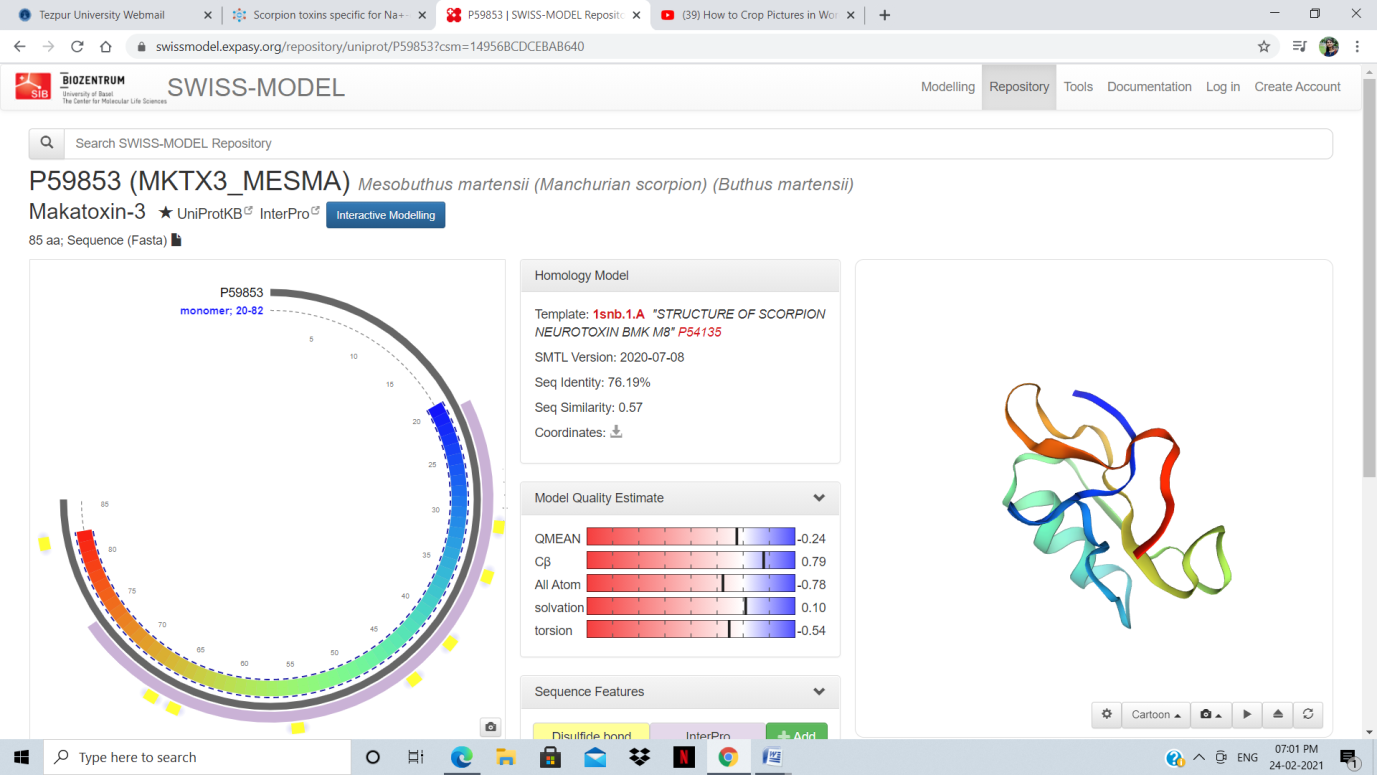 |
| 9 | Serine protease like protein | P0C8M2 | 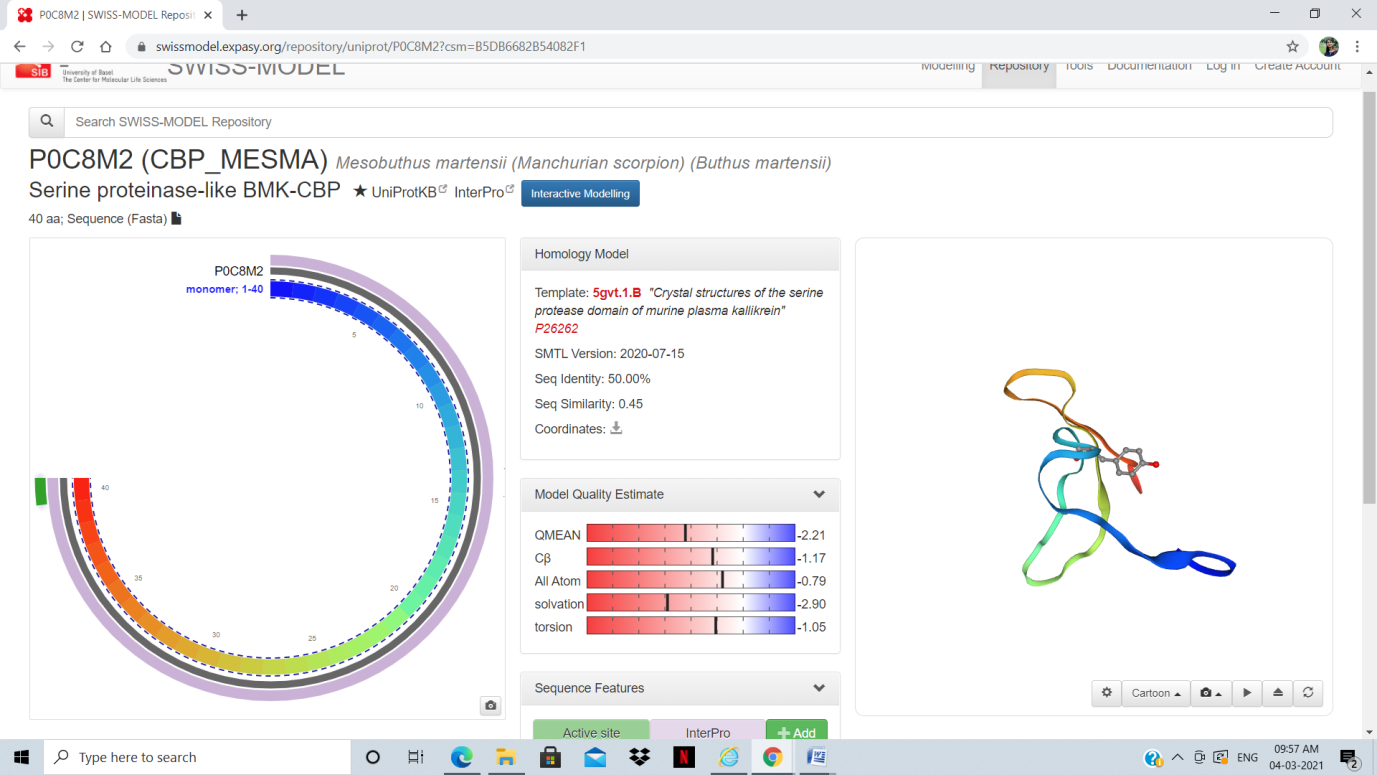 |
| 10 | Serine protease inhibitor | P0DJ47 | 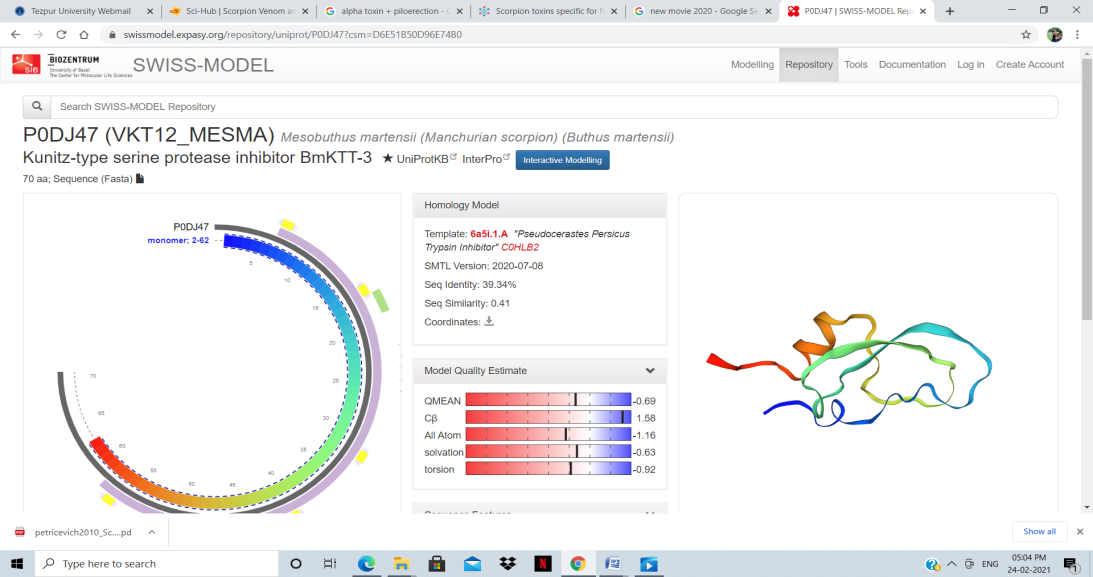 |
| 11 | Antimicrobial peptide |  | Not found |
| 12 | Lipolysis potentiating peptide | Q6WJF5 | 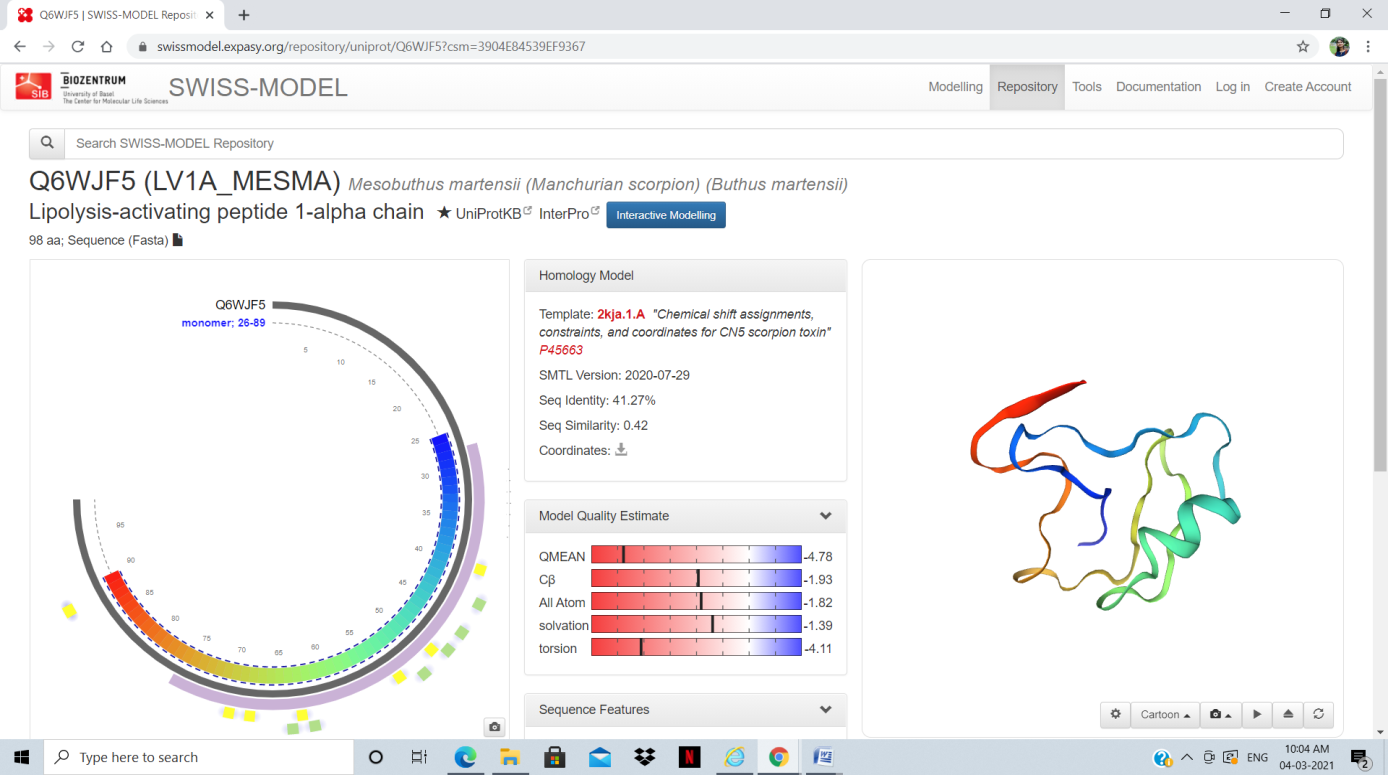 |
| 13 | Parabutoporin | P83312 | Not found |
| 14 | Bradykinin potentiating peptide | Q9TWD3 | Not found |
| 15 | HMG CoA reductase inhibitor | Q95P90 | Not found |

**Supplementary table S3:** Comparative list of the distribution of venom toxins from different species of *Mesobuthus and Heterometrus* throughout the Indian subcontinent.

| **Toxins** | ***Mesobuthus tamulus*** | ***Mesobuthus martensii*** | ***Heterometruslongimanus*** | **References** |
| --- | --- | --- | --- | --- |
| Na^+^ channel toxin | YES | YES | YES | Das et al., 2020; Xu et al, 2014; Bringans et al., 2008 |
| K^+^ channel toxin | YES | YES | YES | Das et al., 2020; Xu et al, 2014; Bringans et al., 2008 |
| Cl^-^ channel toxin | YES | YES | Not known | Das et al., 2020; Xu et al, 2014 |
| Ca^2+^ channel toxin | YES | YES | YES | Das et al., 2020; Xu et al, 2014; Bringans et al., 2008 |
| Hyaluronidase | YES | YES | Not known | Das et al., 2020; Xu et al, 2014 |
| Bukatoxin | YES | Not known | Not known | Das et al., 2020 |
| Makatoxin | YES | YES | Not known | Das et al., 2020; Xu et al, 2014 |
| Serine protease like protein | YES | YES | Not known | Das et al., 2020; Xu et al, 2014 |
| Serine protease inhibitor | YES | YES | Not known | Das et al., 2020; Xu et al, 2014 |
| Antimicrobial peptide | YES | YES | YES | Das et al., 2020; Xu et al, 2014; Bringans et al., 2008 |
| Lipolysis potentiating peptide | YES | YES | Not known | Das et al., 2020; Xu et al, 2014 |
| Parabutoporin | YES | Not known | Not known | Das et al., 2020 |
| Bradykinin potentiating peptide | YES | Not known | Not known | Das et al., 2020 |
| HMG CoA reductase inhibitor | YES | Not known | Not known | Das et al., 2020 |
| Insect toxin | YES | Not known | YES | Das et al., 2020; Wang et al., 2003 |
| Dermonecrotic toxin | Not known | Not known | YES | Bringans et al., 2008 |
